# A Unifying Framework for Reinforcement Learning and Planning

**DOI:** 10.3389/frai.2022.908353

**Published:** 2022-07-11

**Authors:** Thomas M. Moerland, Joost Broekens, Aske Plaat, Catholijn M. Jonker

**Affiliations:** ^1^Leiden Institute of Advanced Computer Science (LIACS), Leiden University, Leiden, Netherlands; ^2^Interactive Intelligence, Delft University of Technology, Delft, Netherlands

**Keywords:** planning, reinforcement learning, model-based reinforcement learning, framework, overview, synthesis

## Abstract

Sequential decision making, commonly formalized as optimization of a Markov Decision Process, is a key challenge in artificial intelligence. Two successful approaches to MDP optimization are *reinforcement learning* and *planning*, which both largely have their own research communities. However, if both research fields solve the same problem, then we might be able to disentangle the common factors in their solution approaches. Therefore, this paper presents a unifying algorithmic framework for reinforcement learning and planning (FRAP), which identifies underlying dimensions on which MDP planning and learning algorithms have to decide. At the end of the paper, we compare a variety of well-known planning, model-free and model-based RL algorithms along these dimensions. Altogether, the framework may help provide deeper insight in the algorithmic design space of planning and reinforcement learning.

## 1. Introduction

Sequential decision making is a key challenge in artificial intelligence (AI) research. The problem, commonly formalized as a Markov Decision Process (MDP) (Bellman, 1954; Puterman, 2014), has been studied in different research fields. The two prime research directions are *reinforcement learning* (RL) (Sutton and Barto, 2018), a subfield of machine learning, and *planning* (also known as *search*), of which the discrete and continuous variants have been studied in the fields of artificial intelligence (Russell and Norvig, 2016) and control theory (Bertsekas, 2012), respectively. Departing from different assumptions both fields have largely developed their own methodology, which has cross-pollinated in the field of *model-based reinforcement learning* (Sutton, 1990; Hamrick, 2019; Moerland et al., 2020a; Plaat et al., 2021).

However, a unified view on both fields, including how their approaches overlap or differ, lacks in literature. For example, the classic AI textbook by Russell and Norvig (2016) discusses (heuristic) search methods in Chapters 3, 4, 10, and 11, while reinforcement learning methodology is separately discussed in Chapter 21. Similarly, the classic RL textbook by Sutton and Barto (2018) does discuss a variety of the topics in our framework, but never summarizes these as a single algorithmic space. Moreover, while the book does extensively discuss the relation between reinforcement learning and dynamic programming methods, it does not focus on the relation with the many other branches of planning literature. Therefore, this paper introduces a Framework for Reinforcement learning and Planning (FRAP) (**Table 2**), which attempts to identify the underlying algorithmic space shared by RL and MDP planning algorithms. We show that a wide range of algorithms, from Q-learning (Watkins and Dayan, 1992) to Dynamic Programming (Bellman, 1954) to A^⋆^ (Hart et al., 1968), fit the framework, simply making different decisions on a number of subdimensions of the framework (**Table 7**).

We need to warn experienced readers that many of the individual topics in the paper will be familiar to them. However, the main contribution of this paper is not the discussion of these ideas themselves, but in the *systematic structuring* of these ideas into a single algorithmic space (Algorithm 1). Experienced readers may therefore skim over some sections more quickly, and only focus on the bigger integrative message. As a second contribution, we hope the paper points researchers from one of both fields toward relevant literature from the other field, thereby stimulating cross-pollination. Third, we note that the framework is equally useful for researchers from model-free RL, since to the best of our knowledge “a framework for reinforcement learning” does not exist in literature either (“a framework for planning” does, see Related Work). Finally, we hope the paper may also serves an educational purpose, for example for students in a University course, by putting algorithms that are often presented in different courses into a single perspective.

**Algorithm 1:**
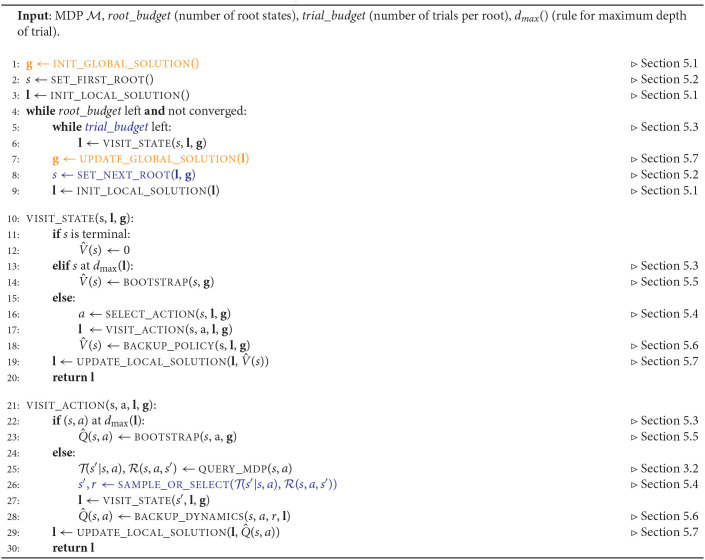
FRAP pseudocode. In planning, there is no global solution, and the orange lines therefore disappear (and **g** therefore drops from all functions as well). In model-free RL there are restrictions on the blue lines: we can only select actions and next states in a single forward trace per root, which indirectly restricts the trial budget per root (to the number of target depths we reweight over within the trace, which is often set to one) and the way we set the next root (which either has to be a next state we reached within the trial or a reset to an initial state of the MDP). In model-based RL, all elements of the framework can be active.

We also need to clearly demarcate what literature we do and do not include. First of all, planning and reinforcement learning are huge research fields, and the present paper is definitely *not* a systematic survey of both fields (which would likely require multiple books, not a single article). Instead, we focus on the core ideas in the joint algorithmic space and discuss characteristic, well-known algorithms to illustrate these key ideas. For the planning side of the literature, we exclusively focus on planning algorithms that search for *optimal behavior* in an MDP formulation, which for example excludes all non-MDP planning methods, as well as “planning as satistifiability” approaches, which attempt to verify whether a path from start to goal exists at all (Kautz et al., 1992, 2006). For the reinforcement learning side of the literature, we do not focus on approaches that treat the MDP formulation as a *black-box optimization problem*, such as evolutionary algorithms (Moriarty et al., 1999), simulated annealing (Atiya et al., 2003) or the cross-entropy method (Mannor et al., 2003). While these approaches can be successful (Salimans et al., 2017), they typically only require access to an evaluation function, and do not use MDP specific characteristics in their solution (on which our framework is built).

The remainder of this article is organized as follows. After discussing Related Work (Section 2), we first formally introduce the MDP optimization setting (Section 3.1), the way we may get access to the MDP (Section 3.2), and give definitions of planning and reinforcement learning (Section 3.3). The next section provides brief overviews of literature in planning (Section 4.1) and reinforcement learning (Section 4.2). Together, Sections 3 and 4 should establish common ground to build the framework upon. The main contribution of this paper, the framework, is presented in Section 5, where we systematically discuss each consideration in the algorithmic space. Finally, Section 6 illustrates the applicability of the framework, by comparing a range of planning and reinforcement learning algorithms along the framework dimensions, and identifying interesting directions for future work.

## 2. Related Work

The basis for a framework approach to planning (and reinforcement learning) is the FIND-and-REVISE scheme by Bonet and Geffner (2003a). FIND-and-REVISE specifies a general procedure for asynchronous value iteration, where we first *find* a new node that requires updating, and subsequently *revise* the value estimate of that node based on interaction with the MDP. Our framework follows as similar pattern, where we repeatedly find a new state (a root that requires updating), find interesting subsequent states to compute an improved value estimate for this state, and subsequently use this estimate to improve the solution. Our framework is also partially inspired by the reinforcement learning textbook of Sutton and Barto (2018), which provides an unified view on the back-up patterns in planning and reinforcement learning (regarding their depth and width), and thereby an integrated view on dynamic programming and reinforcement learning methodology. Similar ideas return in our framework, but we extend them with several additional dimensions, and to a wide variety of other planning literature.

However, the main inspiration of our work is *trial-based heuristic tree search* (THTS) (Keller and Helmert, 2013; Keller, 2015), a framework that subsumed several planning algorithms, like Dynamic Programming (Bellman, 1954), MCTS (Kocsis and Szepesvári, 2006) and heuristic search (Pearl, 1984) methods. THTS shows that a variety of planning algorithms can indeed be unified in the same algorithmic space, which we believe provided a lot of insight in the commonalities of these algorithms. Our present framework can be seen as an extension and modification of these ideas to also incorporate literature from the reinforcement learning community. Compared to THTS, we first of all add several new categories to the framework, such as “solution representation” and “update of the solution,” to accommodate for the various ways in which planning and RL methods differ in the way they store and update the outcome of their back-ups. Second, THTS purely focused on the online planning setting, while we incorporate a new dimension “set root state” that also allows for different prioritization schemes in offline planning and learning. Third, we make several smaller adjustments and extensions, such as splitting up the back-up dimension in several subdimensions, and using a different definition of the concept of a trial (which we define as a single forward sequence of states and actions), which allows us to bound the computational effort per trial. This also leads to a new “budget per root” dimension in the framework, which now specifies the number of trials (width) of the unfolded subtree in the local solution. We nevertheless invite the reader to also read the THTS papers, since they are a useful companion to the present paper.

## 3. Definitions

In sequential decision-making, formalized as Markov Decision Process optimization, we are interested in the following problem: given a (sequence of) state(s), what next action is best to choose, based on the criterion of highest cumulative pay-off in the future. More formally, we aim for *context-dependent action prioritization based on a (discounted) cumulative reward criterion*. This is a core challenge in artificial intelligence research, as it contains the key elements of the world: there is sensory information about the environment (states), we can influence that environment through actions, and there is some notion of what is preferable, now and in the future. The formulation can deal with a wide variety of well-known problem instances, like path planning, robotic manipulation, game playing and autonomous driving.

### 3.1. Markov Decision Process

The formal definition of a *Markov Decision Process* (MDP) (Puterman, 2014) is a tuple $M=\left\{S,A,T,R,\gamma ,p_{0}\left(s\right)\right\}$. The environment consists of a *transition function* T:S×A→p(S) and a *reward function* R:S×A×S→ℝ. At each timestep *t* we observe some state $s_{t}\in S$ and pick an action $a_{t}\in A$. Then, the environment returns a next state $s_{t+1}\sim T\left(s_{t+1}|s_{t},a_{t}\right)$ and associated scalar reward $r_{t}=R\left(s_{t},a_{t},s_{t+1}\right)$. The first state is sampled from the initial state distribution *p*_0_(*s*), while γ ∈ [0, 1] denotes a discount parameter.

The state space can either have an atomic, factorized, or structured form (Russell and Norvig, 2016). *Atomic* state spaces treat each state as a separate, discrete entity, without the specification of any additional relation between states. In contrast, factorized states consist of a vector of attributes, which thereby provide a relation between different states (i.e., the attributes of states may partially overlap). Factorized state spaces allow for *generalization* between states, an important feature of learning algorithms. Finally, *structured* state spaces consist of factorized states with additional structure beyond simple discrete or continuous values, for example in the form of a symbolic language. In this work, we primarily focus on settings with atomic or factorized states.

The agent acts in the environment according to a *policy* π:S→p(A). In the search community, a policy is also known as a *contingency plan* or *strategy* (Russell and Norvig, 2016). By repeatedly selecting actions and transitioning to a next state, we can sample a *trace* through the environment. The *cumulative return* of the trace is denoted by: $J_{t}=\overset{K}{\underset{k=0}{\sum }}{\left(\gamma \right)}^{k}\cdot r_{t+k}$, for a trace of length *K*. For *K* = ∞ we call this the infinite-horizon return. The action-value function *Q*^π^(*s, a*) is defined as the expectation of this cumulative return given a particular policy π:


(1) $$\begin{array}{l} Q^{\pi }(s,a)\;\dot{=}\;E_{\pi ,T}[\sum_{k=0}^{K}\gamma^{k}\cdot r_{t+k}|s_{t}=s,a_{t}=a] \end{array}$$

This equation can be written in a recursive form, better known as the *Bellman equation*:


(2) $$\begin{array}{l} Q^{\pi }(s,a)\;=E_{s^{\prime }\sim T(\cdot |s,a)}\left[ℛ(s,a,s^{\prime })+\gamma E_{a^{\prime }\sim \pi (\cdot |s^{\prime })}[Q^{\pi }(s^{\prime },a^{\prime })]\right] \end{array}$$

Our goal is to find a policy π that maximizes our expected return *Q*^π^(*s, a*):


(3) $$\begin{array}{l} \pi^{⋆}=\underset{\pi }{\arg\;\max\;}Q^{\pi }\left(s,a\right) \end{array}$$

In the planning and control literature, the above problem is typically formulated as a cost *minimization* problem (Bellman, 1957). That formulation is interchangeable with our presentation by negating the reward function. The formulation also contains *stochastic shortest path* (SSP) problems (Bertsekas and Tsitsiklis, 1991), which are a common setting in the planning literature. SSP problems are MDP specifications with negative rewards on all transitions and particular terminal goal states, where we attempt to reach the goal with as little cost as possible. The MDP specification induces a graph, which is in the planning community commonly referred to as an *AND-OR graph*: we repeatedly need to choose between actions (OR), and then take the expectation over the next states (AND). In a search tree these two operations are sometimes referred to as *decision nodes* (OR) and *chance nodes* (AND), respectively.

### 3.2. Access to the MDP Dynamics

A crucial aspect in MDP optimization is the way we can interact with the MDP, i.e., the *type of access* we have to the transition and reward function. We will here focus on the type of access to the transition function, since the type of access to the reward usually mimics the type of access to the transition function. All MDP algorithms at some point *query* the MDP transition function at a particular state-action pair (*s, a*), and get information back about the possible next state(s) *s*′ and associated reward $R\left(s,a,s^{\prime }\right)$. However, there are differences in the *order* in which we can make queries, and in the *type of information* we get back after a query (Kearns et al., 2002; Keller and Helmert, 2013).

**Figure 1:**
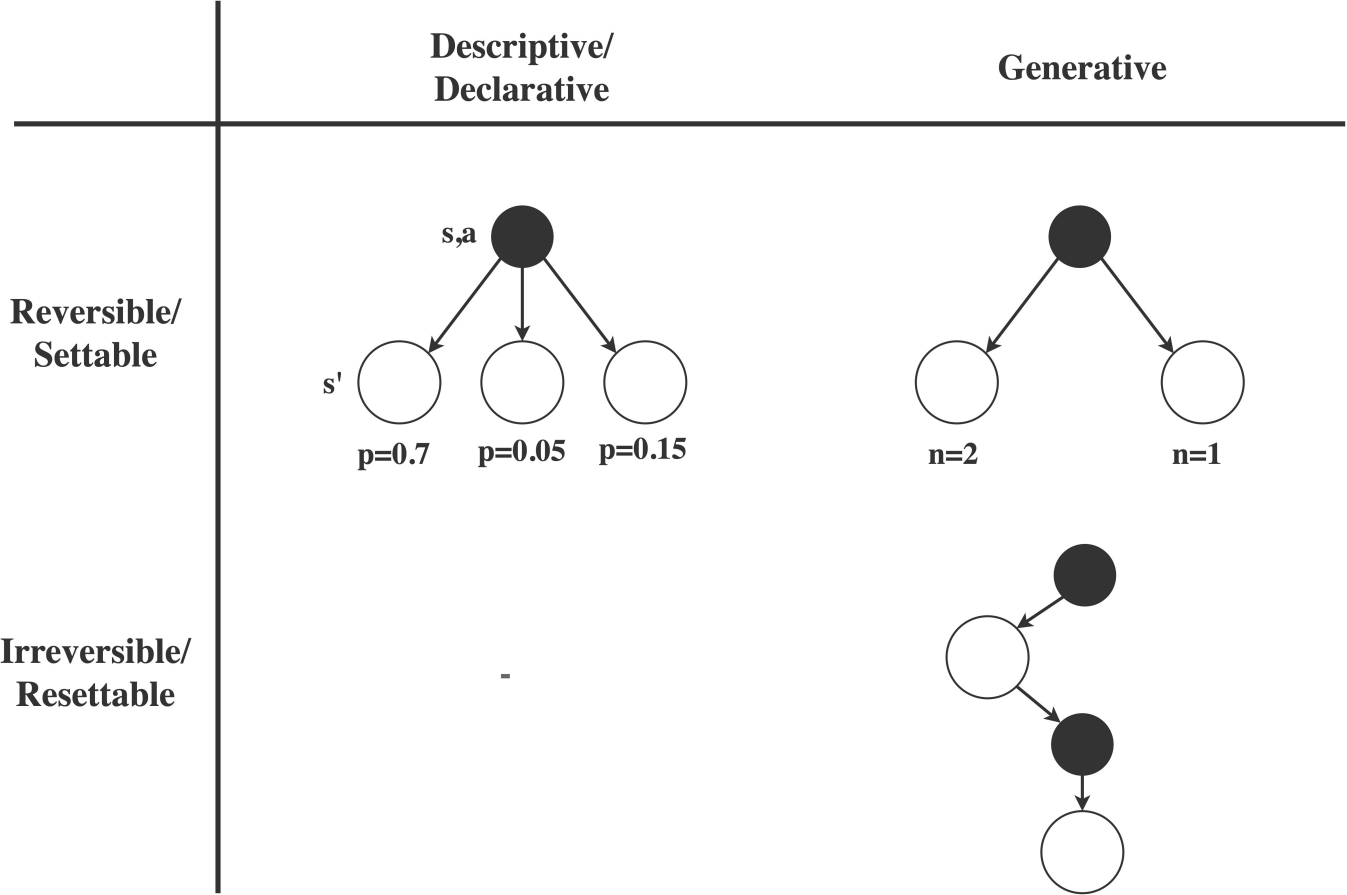
Illustration of different types of access to the MDP transition dynamics. Rows: We may either have *reversible/settable* access to the MDP dynamics, in which case we can query the MDP on any desired state, or *irreversible/resettable* access to the MDP, in which case we have to make the next query at the resulting state, or we can reset to a state from the initial state distribution. Any type of reversible/settable access to the MDP is usually called a (known) *model*. Columns: On each query to the MDP dynamics, we may either get access to the full distribution of possible next states (*descriptive*/*declarative* access), or only get a single sample from this distribution (*generative* access). Note that we could theoretically think of irreversible descriptive access, in which we do see the probabilities but need to continue from the next state, but we are unaware of such a model in practice.

Regarding the first consideration, reinforcement learning methods often assume we need to make our next query at the state that resulted from our last query, i.e., we have to move forward (similar to the way humans interact with the real world). We propose to call this *irreversible* access to the MDP, since we cannot revert a particular action. In practice, RL approaches often assume that we can reset at any particular moment to a state sampled from the initial state distribution, so we may also call this *resettable* access to the MDP. In contrast, planning methods often assume we can query the MDP dynamics in any preferred order of state-action pairs, i.e., we can *set* the query to any state we like. This property also allows us to repeatedly plan forward from the same state (like humans plan in their mind), which we therefore propose to call *reversible* access to the MDP dynamics. The distinction between reversible/settable and irreversible/resettable access is visualized in the rows of Figure 1. Reversible/settable access to the MDP dynamics is usually referred to as a (known) *model*.

*A model is a type of access to the MDP dynamics that can be queried in any preferred order of state-action pairs*.

A second important distinction concerns the type of information we get about the possible next states. A *descriptive/declarative* model provides us the full probabilities of each possible next state, i.e., the entire distribution of $T\left(s^{\prime }|s,a\right)$, which allows us to fully evaluate the expectation over the dynamics in the Bellman equation (Equation 2). In contrast, *generative* access only provides us with a sample from the next state distribution, without access to the true underlying probabilities (we may of course approximate the expectation in Equation (2) through repeated sampling). These two options are displayed in the columns of Figure 1).

Together, the two considerations lead to three types of access to the MDP dynamics, as shown in the cells of Figure 1. Reversible descriptive access (top-left) is for example used in Value Iteration (Bellman, 1957), reversible generative access (top-right) is used in Monte Carlo Tree Search (Kocsis and Szepesvári, 2006), while irreversible generative access (bottom-right) is used in Q-learning (Watkins and Dayan, 1992). The combination of irreversible and descriptive access, in the bottom-left of Figure 1), is theoretically possible, but to our knowledge does not occur in practice. Note that there is also a natural ordering in these types of MDP access: reversible descriptive access gives most information and freedom, followed by reversible generative access (since we can always sample from distributional access), and then followed by irreversible generative access (since we can always restrict the order of sampling ourselves). However, the difficulty to obtain a particular type of access follows the opposite pattern: descriptive models are typically hardest to obtain, while a irreversible generative access is by definition available through real-world interaction.

### 3.3. Definitions of Planning and Reinforcement Learning

We are now ready to give formal definitions of MDP planning and reinforcement learning. While there are various definitions of both fields in literature (Russell and Norvig, 2016; Sutton and Barto, 2018), these are typically not specific enough to discriminate planning from reinforcement learning. One possible distinction is based on the *type of access* to the MDP dynamics: planning approaches had settable/reversible access to the dynamics (“a known model”), while reinforcement learning approaches had irreversible access (“an unknown model”). However, there is a second possible distinction, based on the *coverage or storage of the solution*. This distinction seems known to many researchers, but is seldomly expicitly discussed in research papers. On the one hand, planning methods tend to use *local* solution representations: the solution is only stored temporarily, and usually valid for only a subset of all states (for example repeatedly simulating forward from a current state). In contrast, reinforcement learning approaches tend to use a *global* solution: a permanent storage of the solution which is typically valid for all possible states.

*A local solution temporarily stores solution estimates for a subset of all states*.

*A global solution permanently stores solution estimates for all states*.

The focus of RL methods on global solutions is easy to understand: without a model we cannot repeatedly simulate forward from the same state, and therefore our best bet is to store a solution for all possible states (we can never build a local solution beyond size one, since we have to move forward). The global solutions that we gradually update are typically referred to as *learned* solutions, which connects reinforcement learning to the broader machine learning literature.

Interestingly, our two possible distinctions between planning and reinforcement learning (model vs. no model, and local vs. global solution) do not always agree. For example, both Value Iteration (Bellman, 1966) and AlphaZero (Silver et al., 2018) combine a global solution (which would make it reinforcement learning) with a model (which would make it planning). Indeed, Dynamic Programming has long been considered a bridging technique between planning and reinforcement learning. We propose to solve this issue by considering these borderline cases as *model-based reinforcement learning* (Samuel, 1967; Sutton, 1990; Moerland et al., 2020a), and thereby let the global vs. local distinction dominate.

*Planning is a class of MDP algorithms that 1) use a model and 2) only store a local solution*.

*Reinforcement learning is a class of MDP algorithms that store a global solution*.

The definition of reinforcement learning may then be further partitioned into model-free and model-based RL:

*Model-free reinforcement learning is a class of MDP algorithms that 1) do not use a model, and 2) store a global solution*.

*Model-based reinforcement learning is a class of MDP algorithms that 1) use a model, and 2) store a global solution*.

These definitions are summarized in Table 1. We explicitly introduce these definitions since the boundaries between both fields have generally remained vague, and a clear separation (for example between local and global solutions) will later on be useful in our framework as well.

**Table 1:** Categorization of planning and reinforcement learning, based on 1) the presence of a model (settable/reversible access to the MDP to the MDP dynamics), and 2) the presence of a global/learned solution.

	**Model**	**Global solution**
Planning	+	-
Reinforcement learning	+/-	+
Model-free reinforcement learning	-	+
Model-based reinforcement learning	+	+

## 4. Background

Both planning and reinforcement learning are mature research fields with a large corpus of literature. As mentioned in the Introduction, the intention of this paper is not to provide full surveys of these fields. Instead, the aim of this section is to provide a quick overview of research directions in both fields, pointing into the directions of relevant literature.

### 4.1. Planning

*Planning* (or *search*) is a large research field within artificial intelligence (LaValle, 2006; Russell and Norvig, 2016). A classic approach in MDP planning is *dynamic programming* (DP), of which value iteration (VI) (Bellman, 1966) and policy iteration (PI) (Howard, 1960) are classic examples. DP algorithms sweep through the entire state space, repeatedly solving small subproblems based on the Bellman optimality equation. Dynamic programming is thereby a bridging technique between planning and reinforcement learning (since it combines a model and a global representation of the solution), and would under our definitions be a form of model-based reinforcement learning. While guaranteed to converge on the optimal value function, we typically cannot store the entire solution in tabular form due to the curse of dimensionality (Bellman, 1966). Sometimes tables may be stored more efficiently, for example through binary decision diagrams (BDD) (Akers, 1978; Bryant, 1992), or we can battle the curse of dimensionality through approximate solutions (Powell, 2007; Bertsekas, 2011), which we further discuss in the section on reinforcement learning.

Most planning literature has focused on local solution derived from traces sampled from some start state, which are often represented as *trees* or *graphs*. Historically this starts with research on *uninformed search*, which studied the order of node expansion in a search tree, like *breadth-first search* (BFS) (Moore, 1959), *depth-first search* (Tarjan, 1972), and *iterative deepening* (Slate and Atkin, 1983). However, most planning algorithms follow a pattern of *best-first search*, where we next expand the node which currently seems most promising. An early example is Dijkstra's algorithm (Dijkstra, 1959), which next expands the node with the current lowest path cost. Dijkstra also introduced the notions of a *frontier* (or open set), which is the set of states on the border of the planning tree/graph that are still candidate for expansion, and of an *explored states* (or closed set), which is the set of states that have already been expanded. By tracking a frontier and explored set we turn a tree search into a graph search, since it prevents the further expansion of *redundant* paths (multiple action sequences leading to the same state).

We may further improve planning performance through the use of *heuristics* (Simon and Newell, 1958), which in planning are often functions that provide a quick, optimistic estimate of the value of a particular state. When we apply best-first search to the sum of the path cost and admissible heuristic, we arrive at the well-known search algorithm A^⋆^ (Hart et al., 1968), which is applicable to deterministic domains. The same approach was extended to the stochastic MDP setting as AO^⋆^ (Pohl, 1970; Nilsson, 1971). Another successful idea in the (heuristic) planning literature is the use of *labeling* to mark a particular state as solved (not requiring further expansion) when its value estimate is guaranteed to have converged (which happens when the state is either terminal or all of its children have been solved). Labeling can be challenging due to the potential presence of loops (which we can expand indefinitely), for which LAO^⋆^ (Hansen and Zilberstein, 2001) further extends the AO^⋆^ algorithm. A survey of heuristic search is provided by Pearl (1984), while Kanal and Kumar (2012) discuss the relation of these methods to *branch-and-bound* search, which has been popular in operations research.

A bridging algorithm from the planning to the learning community was *learning real-time* A^⋆^ (LRTA^⋆^) (Korf, 1990), which started to incorporate learning methodology in planning methods (and was as such one of the first model-based RL papers). This approach was later extended to the MDP setting as Real-time Dynamic Programming (RTDP) (Barto et al., 1995), which performs DP updates on traces sampled from a start state distribution. *Labeled-RTDP* (Bonet and Geffner, 2003b) extends RTDP through a labeling mechanism for solved states, with further improvements of RTDP provided by McMahan et al. (2005), Smith and Simmons (2006), and Sanner et al. (2009).

Many planning algorithms suffer from high-memory requirements, since it is typically infeasible to store all possible states in memory. Several research lines have therefore investigated planning algorithms that have reduced memory requirements. Some well-known examples are *iterative deepening* depth-first search (Slate and Atkin, 1983), iterative deepening A^⋆^ (Korf, 1985), Simplified Memory-Bounded A^⋆^ (SMA^⋆^) (Russell, 1992) and recursive best-first search (RBFS) (Korf, 1993). For a more extensive discussion of (heuristic) MDP planning methods we refer the reader to Kolobov (2012) and Geffner and Bonet (2013).

A different branch in planning research estimates action values based on statistical sampling techniques, better known as *sample-based planning*. A classic approach is *Monte Carlo search* (MCS) (Tesauro and Galperin, 1997), in which we sample a number of traces for each currently available action and estimate their value as the mean return of these traces. Sample-based planning was further extended to *sparse sampling* (Kearns et al., 2002), which formed the basis for *Monte Carlo Tree Search* (MCTS) (Coulom, 2006; Kocsis and Szepesvári, 2006; Browne et al., 2012). While MCS only tracks statistics at the root of the tree search, MCTS recursively applies the same principle at deeper levels of the tree as well. Exploration and exploitation within the tree are typically based on variants of the upper confidence bounds (UCB) rule (Auer et al., 2002). MCTS for example showed early success in the game of Go (Gelly and Wang, 2006). In the control community, there is a second branch of sample-based planning known as *rapidly-exploring random trees* (RRTs) (LaValle, 1998). In contrast to MCTS, which samples in action space to construct a tree, RRTs sample in state space and try to find an action that connects the new sampled state to the existing explicit tree in memory.

Planning in continuous state and actions spaces, like in robotics, is typically referred to as *optimal control* (Lewis et al., 2012; Levine, 2018). Here, dynamics functions are often smooth and differentiable, and many algorithms therefore use a form of *gradient-based planning*. In this case, we directly optimize the policy for the cumulative reward objective by differentiating through the dynamics function. When the dynamics model is linear and the reward function quadratic, the solution is actually available in analytical form, better known as the linear-quadratic regulator (LQR) (Anderson and Moore, 2007). In practice, dynamics are often not linear, but this can be partly mitigated by repeatedly linearizing the dynamics around the current state [known as iterative LQR (iLQR) Todorov and Li, 2005]. In the RL community, gradient-based planning is often referred to as *value gradients* (Heess et al., 2015). Alternatively, we can also write the MDP problem as a non-linear programming problem (i.e., take the more black-box optimization approach), where the dynamics function for example enters as a constraint, better known as *direct optimal control* (Bock and Plitt, 1984). Another research line treats planning as probabilistic inference (Toussaint, 2009; Botvinick and Toussaint, 2012; Kappen et al., 2012), where we construct message-passing algorithms to infer which actions would lead to receiving a final reward.

A popular approach in the control community is *model predictive control* (MPC) (Morari and Lee, 1999), also known as *receding-horizon control* (Mayne and Michalska, 1990), where we optimize for an action up to a certain lookahead depth, execute the best action from the plan, and then re-plan from the resulting next state (i.e., we never optimize for the full MDP horizon). Such interleaving of planning and acting (McDermott, 1978) is in the planning community often referred to as *decision-time* planning or *online* planning, where we directly need to find an action for a current state. In contrast, *background* or *offline* planning (Sutton and Barto, 2018) uses planning operations to improve the solution for a variety of states, for example stored in a global solution.

### 4.2. Reinforcement Learning

Reinforcement learning (RL) (Barto et al., 1983; Wiering and Van Otterlo, 2012; Sutton and Barto, 2018) is a large research field within machine learning. While the planning literature is mostly organized in sub-disciplines (as discussed above), RL literature can best be covered through the range of subtopics within algorithms that have been studied. A central idea in RL is the use of *bootstrapping* (Sutton, 1988), where we plug in a *learned* value estimate to improve the estimate of a state that precedes it. Literature has focused on the way we can construct these bootstrap estimates, for example distinguishing between *on-policy* (Rummery and Niranjan, 1994) and *off-policy* back-ups (Watkins and Dayan, 1992). The depth of the back-up has also received much attention in RL, where estimates of different depths can for example be combined through *eligibility traces* (Singh and Sutton, 1996). We can also use multi-step methods in the off-policy setting through the use of importance sampling, where we generally reweight the back-up contribution of the next step by its probability under the optimal policy. Examples in this direction are the Tree-backup [TB(λ)] algorithm (Precup, 2000) and Retrace(λ) (Munos et al., 2016).

Reinforcement learning research has also focused on direct specification of the solution, in the form of a policy function. An important result in this direction is the *policy gradient theorem* (Williams, 1992; Sutton et al., 2000; Sutton and Barto, 2018), which specifies an unbiased estimate of the gradient of the objective with respect to policy parameters. Policy search methods can be stabilized in various ways (Schulman et al., 2015, 2017), can be integrated with (gradient-based) planning (Deisenroth and Rasmussen, 2011; Levine and Koltun, 2013), and have for example shown much success in robotics (Deisenroth et al., 2013). Note that policy search can also be approached in a gradient-free way, for example through evolutionary strategies (Moriarty et al., 1999; Whiteson and Stone, 2006), including the successful *cross-entropy method* (CEM) (Mannor et al., 2003).

A central theme in reinforcement learning research is the use of supervised learning methods to *approximate* the solution, which allows information to *generalize* between similar states (and in larger problems allow a global solution to fit in memory). Early results on function approximation include tile coding (Sutton, 1996) and linear approximation (Bradtke and Barto, 1996), while state-of-the-art results are achieved by the use of deep neural networks (Goodfellow et al., 2016), whose application to RL was pioneerd by Mnih et al. (2015). Surveys of deep reinforcement learning are provided by François-Lavet et al. (2018) and Arulkumaran et al. (2017).

Another fundamental theme in RL research is the balance between exploration and exploitation. Random perturbation approaches include ϵ-greedy and Boltzmann exploration (Sutton and Barto, 2018), while other approaches, such as confidence bounds (Kaelbling, 1993) and Thompson sampling (Thompson, 1933), leverage the uncertainty in an action value estimate. Another large branch in RL exploration research is *intrinsic motivation* (Chentanez et al., 2005), which explores based on concepts like curiosity (Schmidhuber, 1991), novelty, and model uncertainty (Guez et al., 2012).

Reinforcement learning and planning have been combined in the field of model-based reinforcement learning (Hester and Stone, 2012; Moerland et al., 2020a). In the RL community, this idea started with *Dyna* (Sutton, 1990), which uses sampled data (from an irreversible environment) to learn a reversible dynamics model, and subsequently makes planning updates over this learning model to further improve the value function. Successful model-based RL algorithms include AlphaZero (Silver et al., 2018), which set superhuman performance in Go, Chess and Shogi, and Guided Policy Search (Levine and Koltun, 2013), which was successful in robotics tasks. We can also use a learned model for gradient-based policy updates, as for example done in PILCO (Deisenroth and Rasmussen, 2011), while a learned backward model allows us to more quickly spread new information over the state space [known as *prioritized sweeping* (PS) Moore and Atkeson, 1993]. A full survey of model-based reinforcement learning is provided by Moerland et al. (2020a).

Reinforcement learning research is also organized around a variety of subtopics, such as hierarchical/temporal abstraction (Barto and Mahadevan, 2003), goal setting and generalization over goals (Schaul et al., 2015), transfer between tasks (Taylor and Stone, 2009), and multi-agent reinforcement learning (Busoniu et al., 2008). While these topics are all important, our framework solely focuses on a single agent in a single MDP optimization task. However, note that many of these topics are complementary to our framework (i.e., they could further extend it). For example, we may discover higher-level actions (hierarchical RL) to define a new, more abstract MPD, in which all of the principles of our framework are again applicable.

To summarize, this section covered some important research directions within planning and reinforcement learning. Our treatment was of course superficial, and by no means covered all relevant literature from both fields. Nevertheless, it does provide common ground on the type of literature we consider for our framework. In the next section, we will try to organize the ideas from both fields into a single framework.

## 5. Framework

**Figure 2:**
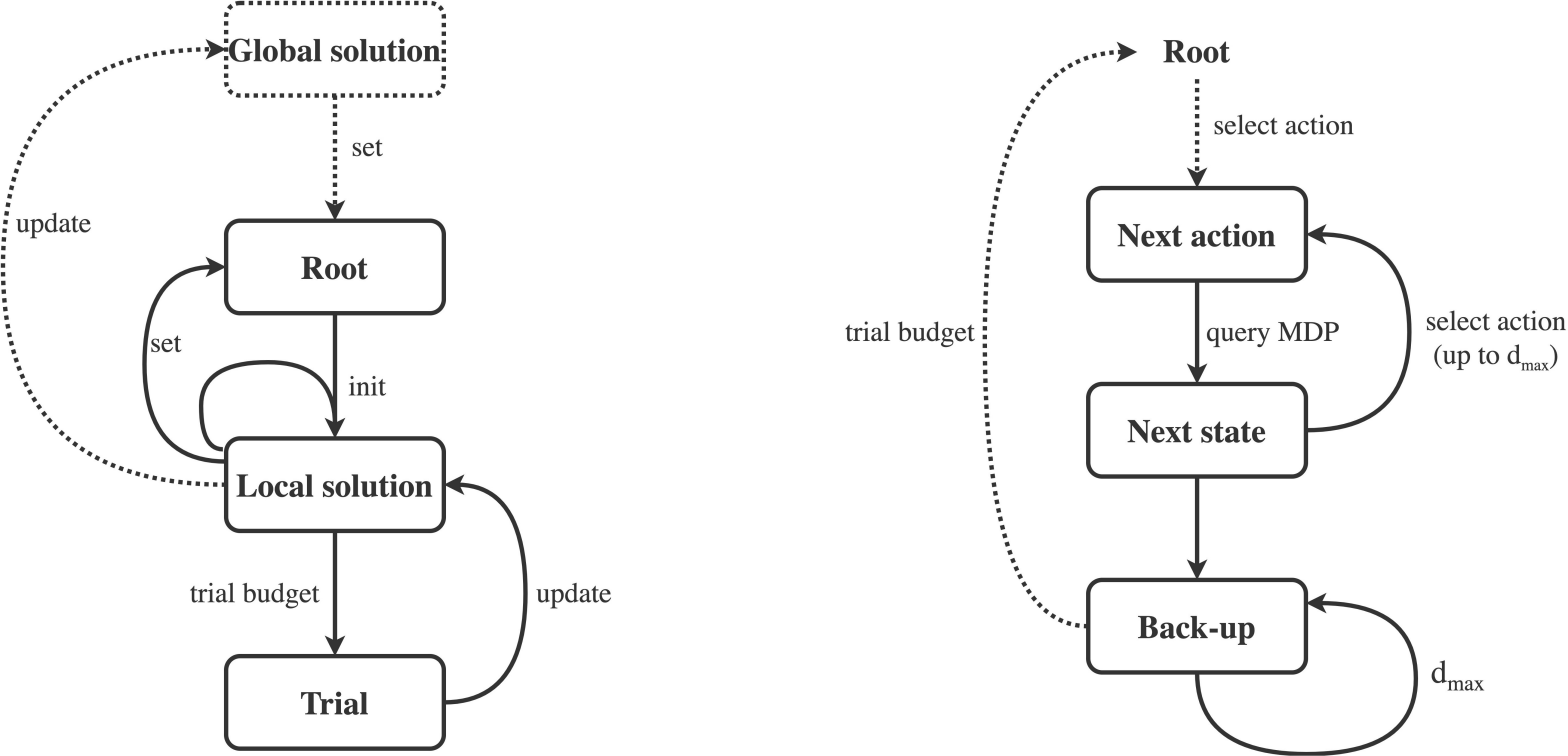
Graphical illustration of framework (Algorithm 1). Left: Algorithm outer loop (Algorithm 1, line 4), illustrating the interplay of global and local solutions with trials. After possibly initializing a global solution, we repeatedly fix a new root state for which we want to improve our solution. Then, we initialize a new local solution for the particular root, and make one or multiple trials (trial budget), where each trial updates the local solution. After the budget is expanded, we may use the local solution to update the global solution and/or set the next root state and/or reuse information for the next local solution. The process then repeats with setting a new root, possible based on the global and/or local solution. Right: Algorithm inner loop (Algorithm 1, line 5), illustrating an individual trial. A trial starts from a root node, from which we repeatedly select actions, query the MDP at the specific state-action pair, and then transition to a next state. We repeat this process *d*_max_ times, after which we start the back-up phase, consisting of *d*_max_ back-ups. When budget is available, we start another trial from the same root node.

We will now introduce the Framework for Reinforcement Learning and Planning (FRAP). Pseudocode for the framework is provided in Algorithm 1, while all individual dimensions are summarized in Table 2. We will first cover the high-level intuition of the framework, as visualized in Figure 2. FRAP centers around the notion of *root states* and *trials*.

*A root state is a state for which we attempt to improve the solution estimate*.

*A trial is a sequence of forward actions and next states from a root state, which is used to compute an estimate of the cumulative reward from the root state*.

**Table 2:** Overview of dimensions in the Framework for Reinforcement learning and Planning (FRAP).

**Dimension**	**Consideration**	**Choices**
		
1. Solution (Section 5.1)	- Coverage	Global, local
	- Type	(Goal-conditioned) value, (goal-conditioned) policy, counts,…
	- Method	Param. tabular, param. approximate, non/semi-parametric
	- Initialization	Uniform, random, optimistic, expert
		
2. Set root state (Section 5.2)	- Selection	Ordered, initial state, forward sampling, backward sampling, previously visited
		
3. Budget per root (Section 5.3)	- Number of trials (width)	1, *n*, convergence, ∞
	- Depth per trial (*d*_max_)	1, *n*, adaptive, ∞
		
4. Selection in trial (Section 5.4)	- Next action	Ordered, greedy (with heuristic), value-based perturbation (random, means, uncertainty), state-based perturbation (knowledge-based IM, competence-based IM)
	- Next state	Sample, ordered
		
5. Bootstrap (Section 5.5)	- Location	State, state-action
	- Type	Learned, heuristic
		
6. Back-up (Section 5.6)	- Back-up policy	Behavioral policy, greedy/max, other policy...
	- Policy expectation	Sample/partial, expected/full
	- Dynamics expectation	Sample/partial, expected/full
	- Additional characteristics	Explored states, convergence label, counts, uncertainty, return distribution
		
7. Update (Section 5.7)	- Loss/objective	Squared loss, policy gradient, value gradient, cross-entropy, etc.
	- Learning rate	Step (η fixed), Replace (η = 1.0 on table), Average (η = 1/*n* on table), Eligibility (η = (1−λ)·λ^(*d*−1)^), Adaptive (trust region), etc.

*Examples for several algorithms are shown in Table 7. IM, Intrinsic Motivation*.

The central idea of FRAP is that all planning and reinforcement learning algorithms repeatedly 1) fix root states, 2) make trials from these root states, 3) improve their solution based on the outcome of these trials, and 4) use this improved solution to better direct new trials and better set new root states. FRAP therefore consists of an *outer loop* (the while loop on Algorithm 1, line 4), in which we repeatedly set new root states, and an *inner loop* (the while loop on Algorithm 1, line 5), in which we (repeatedly) make trials from the current root state to update our solution. We will briefly discuss both loops.

A schematic illustration of the outer loop is shown on the left side of Figure 2. The algorithm starts by potentially initializing a global solution (for all states), and subsequently fixing a new root state. Then, we initialize a local solution for the particular root, and start making trials from the root, which each update the local solution. When we run out of trial budget for this root, we may use the local solution to update the global solution (when used). Afterwards, we fix a next root state, and initialize a new local solution, in which we may reuse information from the last local solution (Algorithm 1, line 9). The outer loop then repeats for the new root state.

The inner loop of FRAP consists of trials, and is schematically visualized on the right of Figure 2. A trial starts from the root node, and consists of a forward sequence of actions and resulting next states and rewards, which are obtained from *queries* to the MDP dynamics. This process repeats *d*_max_ times, where the specification of *d*_max_ depends on the local solution and differs between algorithms. The forward phase of the trial then halts, after which we possibly *bootstrap* to estimate the remaining expected return from the leaf state, without further unfolding the trial. Then, the trial proceeds with a sequence of *one-step back-ups*, which process the acquired information from the forward phase. We repeat the trial process until we run out of budget, after which we fix a new root state (Algorithm 1, line 8).

Action selection in FRAP not only happens within the trial (Algorithm 1, line 16), but is in many algorithms also part of next root selection (Algorithm 1, line 8). It is important to mention that in the case of model-free RL, where we have irreversible access to the MDP dynamics, these two action selection moments are actually equal by definition. For example, a model-free RL agent may fix a root, sample a trial from this root, and use it to update the global solution. However, because the environment is irreversible, the next root selection has to use the same action and resulting next state as was taken within the trial. Model-free RL agents therefore have some specific restrictions in the FRAP pseudocode, as illustrated on the blue lines of Algorithm 1 (the trial budget per root is for example also by definition equal to one).

FRAP is therefore really a conceptual framework, and practical implementations may differ from the pseudocode in Algorithm 1. For example, many planning methods store an explicit frontier, i.e., the set of nodes that are candidate for expansion. Practical implementations would directly jump to the frontier, and not first traverse the known part of the tree from the root, as happens in each trial of Algorithm 1. However, it is conceptually useful to still think of these forward steps, since they will be part of the back-up phase (we are eventually looking for a good decision at the root). Another example would be a model-free RL agent that uses a Monte Carlo return estimate. Practical implementations may sample a full episode, compute the cumulative reward starting from each state in the episode, and jointly update the solution for all these states. However, conceptually every state in the episode has then been a root state once, for which we compute an estimate. In FRAP, we would therefore see this as sampling the actual episode only once from the first root, store it in the local solution, and then repeatedly set new roots along the states in the episode, where we keep reusing the local solution from the last root (Algorithm 1 line 9). In summary, all algorithms conceptually fit FRAP, since they all fix root states for which they compute improved estimates of the cumulative return and solution, but some algorithms may take implementation shortcuts.

We are now ready to discuss the individual dimensions of the framework, i.e., describe the possible choices on each of the lines in Algorithm 1. These dimensions are: how to *represent* the solution, how to *set the next root state*, which *trial budget* to allocate per root state, how to *select* actions and next states within a trial, how to *back-up* information obtained from the trial, and how to *update* the local and global solution based on these back-up estimates. The considerations of FRAP are summarized in Table 2, while the comments on the right side of Algorithm 1 indicate to which lines each dimension is applicable.

### 5.1. Solution Representation

We first of all have to decide how we will represent the solution to our problem. The top row of Table 2 shows the four relevant considerations: the coverage of our solution, the type of function we will represent, the method we use to represent this function, and the way we initialize the chosen method. The first item distinguishes between *local/partial* (for a subset of states) and *global* (for all states) solutions, a topic which we already extensively discussed in Section 3.3. Note that FRAP *always* builds a local solution: even a single episode of a model-free RL algorithm is considered a local solution that estimates the value of states in the trace. A local solution therefore aggregates information from one or more trials, which may then itself be used to update a global solution (when we use one) (Algorithm 1, line 1).

For both local and global solutions we next need to decide what type of function to represent. The most common choices are to represent the solution as a *value* function V:S→ℝ, *state-action value* function Q:S×A→ℝ, or *policy* function π:S→p(A). Some algorithms combine value and policy solutions, better known as *actor-critic* algorithms (Konda and Tsitsiklis, 1999). We may also store the *uncertainty* around value estimates (Osband et al., 2016; Moerland et al., 2017), for example using *counts* (Kocsis and Szepesvári, 2006), or through convergence labels that mark a particular value estimate as solved (Nilsson, 1971; Bonet and Geffner, 2003b). Some methods also store the entire distribution of returns (Bellemare et al., 2017; Moerland et al., 2018), or condition their solution on a particular goal (Schaul et al., 2015) (i.e., store a solution for multiple reward functions).

After deciding on the type of function to represent, we next need to specify the representation method. This is actually a supervised learning question, which we can largely break up in *parametric* and *non-parametric* approaches. *Parametric tabular* representations use a unique parameter for the solution at each state-action pair, which is for example used in the local solution of a graph search, or in the global solution of a tabular RL algorithm. For high-dimensional problems, we typically need to use *parametric approximate* representations, such as (deep) neural networks (Rumelhart et al., 1986; Goodfellow et al., 2016). Apart from reduced memory requirement, a major benefit of approximate representations it their ability to *generalize* over the input space, and thereby make predictions for state-actions that have not been observed yet. However, the individual predictions of approximate methods may contain errors, and there are indications that the combination of tabular and approximate representations may provide the best of both worlds (Silver et al., 2017; Wang et al., 2019; Moerland et al., 2020b). Alternatively, we may also store the solution in a *non-parametric* way, where we simply store exact sampled traces (e.g., a search tree that does not aggregate over different traces), or *semi-parametric* way (Graves et al., 2016), where we may optimize a neural network to write to and read to a table (Blundell et al., 2016; Pritzel et al., 2017), sometimes referred to as *episodic memory* (Gershman and Daw, 2017).

Finally, we also need to initialize our solution representation. Tabular representations are often *uniformly* initialized, for example setting all initial estimates to 0. Approximate representations are often *randomly* initialized, which provides the tie breaking necessary for gradient-based updating. Some approaches use initialization to guide exploration, either through *optimistic initialization* (when a state has not been visited yet, we consider its value estimate to be high) (Bertsekas and Tsitsiklis, 1996) or *expert initialization* (where we use imitation learning from (human) expert demonstrations to initialize the solution) (Hussein et al., 2017). We will further discuss exploration methods in Section 5.4.

An overview of our notation for the different local/global and tabular/approximate solution types is shown in Table 3. We will denote *local* estimates with superscript **l**, e.g., *V*^**l**^(*s*) or *Q*^**l**^(*s, a*), and *global* solutions with superscript **g**, e.g., *V*^**g**^(*s*), *Q*^**g**^(*s, a*) or π^**g**^(*a*|*s*). In practice, only global solutions are learned in approximate form, which we indicate with a subscript θ (for parameters θ).

**Table 3:** Overview of notation.

	**Back-up estimate**	**Local solution**	**Global solution**
Tabular	$\hat{V}\left(s\right)$, $\hat{Q}\left(s,a\right)$	*V*^**l**^(*s*), *Q*^**l**^(*s, a*)	*V*^**g**^(*s*), *Q*^**g**^(*s, a*), π^**g**^(*a*|*s*)
Approximate	(-)	(-)	$V_{\theta }^{\text{g}}\left(s\right)$, $Q_{\theta }^{\text{g}}\left(s,a\right)$, $\pi_{\theta }^{\text{g}}\left(a|s\right)$

*Each trial provides new back-up estimates $\hat{V}\left(s\right)$ and $\hat{Q}\left(s,a\right)$ at the states and actions that appear in the trial. These estimates are aggregated in the local solution V^**l**^(s) and Q^**l**^(s, a) (i.e., the local solution can be influenced by multiple trials). The local solution may itself be used to update the global solution V^**g**^(s), Q^**g**^(s, a) and/or π^**g**^(a|s). When the global solution is stored in approximate form (which is often the case), we denote them by $V_{\theta }^{\text{g}}\left(s\right)$, $Q_{\theta }^{\text{g}}\left(s,a\right)$ and/or $\pi_{\theta }^{\text{g}}\left(a|s\right)$ (where θ denotes the parameters of the approximation). Back-up estimates and local solutions are in practice never represented in approximate form*.

As you will notice, Table 3 contains a separate entry for the *back-up estimate*, $\hat{V}\left(s\right)$ or $\hat{Q}\left(s,a\right)$, which are formed during every trial. Especially researchers from a planning background may find this confusing, since in many algorithms the back-up estimate and local solution are actually the same. However, we should consider these two different quantities, for two reasons. First of all, in some algorithms, like the roll-out phase of MCTS, we do make additional MDP queries (the trial continues) and back-ups, but the back-up estimate from the last part of the trial is never stored in the local solution (the local solution expands with only one new node per trial). Second, many algorithms use their local solution to *aggregate* cumulative reward estimates from different depths, which is for example used in eligibility traces (Sutton and Barto, 2018). For our conceptual framework, we therefore consider each cumulative reward estimate the result of a single trial, and the local solution may combine the estimate of trials in multiple ways. We will discuss ways to aggregate back-up estimates into the local solution in Section 5.7.

### 5.2. Set a Root State

**Figure 3:**
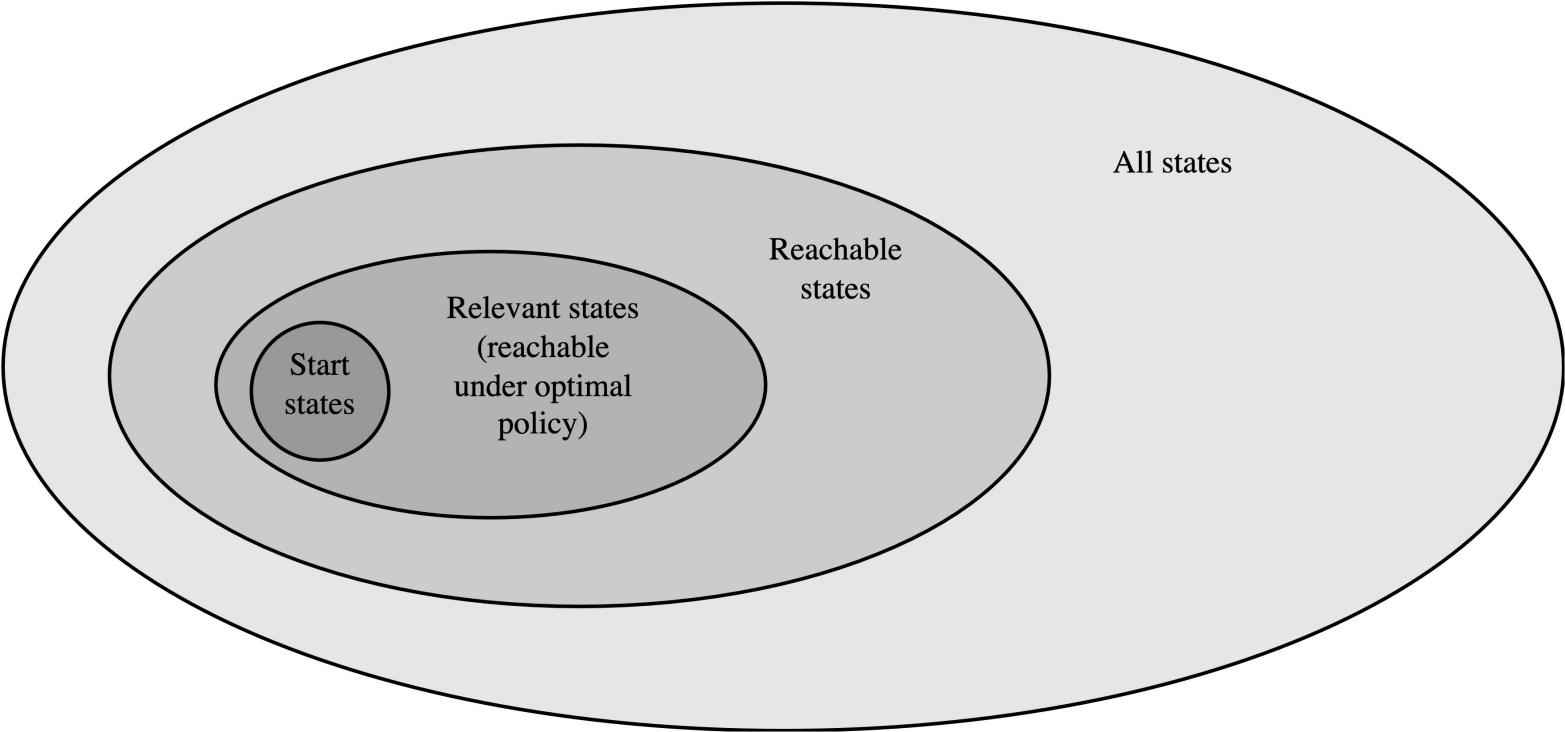
Venn diagram of total state space. Only a subset of the entire state space is *reachable* from the start state under *any policy*. An even smaller subset of the reachable set is eventually *relevant*, in the sense that they are reachable from the start state under the *optimal policy*. Finally, a subset of the relevant state are of course all start states. Figure extended from Sutton and Barto (2018).

The next consideration in our framework is the selection of a root state (Algorithm 1, line 2 and 8), for which we will attempt to improve our solution (by computing a new value estimate). The main considerations are listed in the second row of Table 2. A first approach is to select a state from the state space in an *ordered* way, for a example by sweeping through all possible states (Howard, 1960; Bellman, 1966). A major downside of this approach is that many states in the state space are often not even reachable from the start state (Figure 3), and we may spend much computational effort on states that will never be part of the practical solution.

When the MDP definition includes the notion of a *start state distribution*, this information may be utilized to improve our selection of root states, by only sampling root states on traces from the start. This ensures that new roots are always reachable, which may strongly reduce the number of states we will update in practice (illustrated in Figure 3). In Table 2, we list this as the *forward sampling* approach to selecting new root states. Note that this generally also involves an action selection question (in which direction do we set the next root), which we will discuss in Section 5.4.

The next option is to select new root states in the reverse direction, i.e., through backward sampling (instead of forward sampling). This approach does require a *backwards model* *p*(*s, a*|*s*′), which specifies the possible state-action pairs (*s, a*) that may lead to a next state *s*′. The main idea is to set next root states at the possible precursor states of a state whose value has just changed much, better known as *prioritized sweeping* (Moore and Atkeson, 1993). We thereby focus our update budget on regions of the state space that likely need updating, which may speed-up convergence. Similar ideas have been studied in the planning community as *backward search* or *regression search* (Nilsson, 1982; Bonet and Geffner, 2001; Alcázar et al., 2013), which makes prioritized sweeping an interleaved form of forward and backward search.

Finally, we do not always need to select the next root state from the current trace. For example, we may track the set of *previously visited states*, and select our next root from this set. This approach, which is for example part of Dyna (Sutton, 1990), gives greater freedom in the order of root states, while it still ensures that we only update reachable states. To summarize, we need to decide on a way to set root states, which may for example be done in an ordered way, through forward sampling, through backward sampling, or by selecting previously visited states (Table 2, second row).

### 5.3. Budget per Root

**Figure 4:**
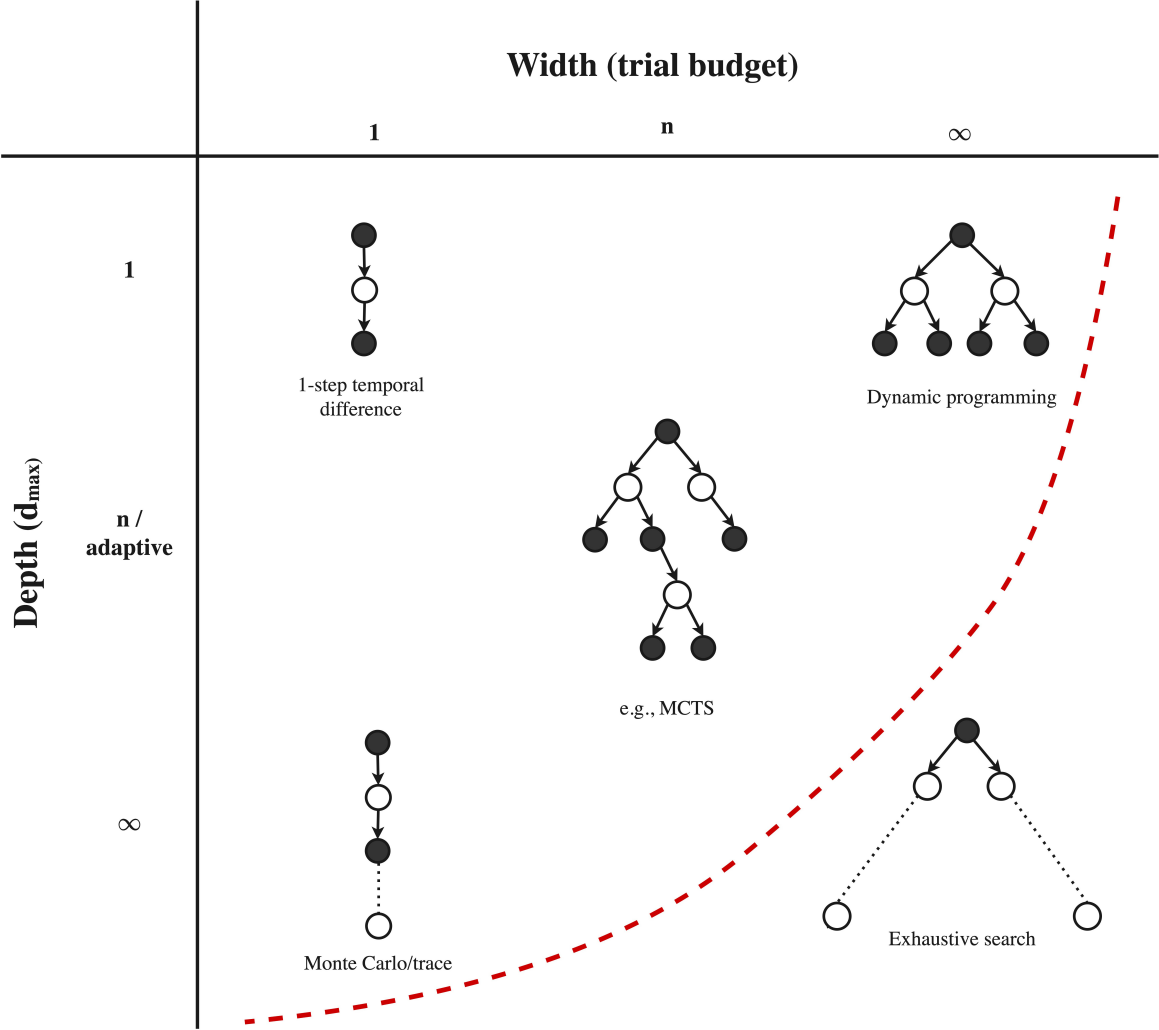
Possible combinations of width (trial budget) and depth (*d*_max_) per trial from a root state. Practical algorithms reside somewhere left of the left dotted line, since full with combined with full depth (exhaustive search) is not feasible in larger problems. Figure extended from Sutton and Barto (2018).

After we fixed a root state (a state for which we will attempt to improve the solution), we need to decide on 1) the number of trials from the particular root (Algorithm 1, line 5), and 2) when a trial itself will end, i.e., the depth *d*_max_ of each forward trial (Algorithm 1, line 13 & 22). These possible choices on each of these two considerations are listed in the third row of Table 2. Note that since every trial consists of a single forward beam, the total number of trials is actually a good measure of the total width of the local solution (**Figure 6**). The joint space of both considerations is visualized in Figure 4, which we will discuss below.

Regarding the *trial budget per root state*, a first possible choice is to only run a single trial. This choice is characteristic for model-free RL algorithms (Sutton and Barto, 2018). Algorithms that have access to a model may also run multiple trials per root state. This budget can for example be specified as a fixed hyperparameter, as is often the choice in MCTS (Browne et al., 2012). When we interact with a real-world environment, the trial budget may actually be enforced by the time until the next decision is required. In the planning community, this is referred to as *decision time planning* or *online planning*. In offline approaches, we may also provide an adaptive trial budget, for example until some convergence criterion is met (often in combination with an admissible heuristic, which may reduce the required number of trials to convergence a lot) (Nilsson, 1971; Hansen and Zilberstein, 2001; Bonet and Geffner, 2003b). Finally, we may also specify an infinite trial budget, i.e., we will repeat trials until all possible sequences (for the specified depth) have been expanded.

The second decision involves the *depth* of each individual trial. A first option is to use a trial depth of one, which is for example part of value/policy iteration (Bellman, 1966) and temporal difference learning (Sutton, 1988; Watkins and Dayan, 1992; Rummery and Niranjan, 1994). We may also specify a fixed multi-step depth, which is the case for *n*-step methods, or specify a full depth (∞), in which case we unroll the trail until a terminal state is reached (in practice we often still limit the trial by a large depth). The latter is also known as a *Monte Carlo roll-out*, which is for example used in MCTS. Finally, many algorithms make use of an *adaptive* trial depth, which depends on the current local solution (i.e., note that *d*_max_(**l**) depends on **l** in Algorithm 1, lines 13 and 22). For example, several (heuristic) planning algorithms terminate a trial once we reach a state or action that did not appear in our current local solution yet (Hart et al., 1968; Nilsson, 1971). As another example, we may terminate a trial once it reaches a state in the explored set or makes a cycle to a duplicate state, which are also examples of an adaptive *d*_max_(**l**). To summarize, the trial budget and depth of each trial are important considerations in all planning and RL algorithms.

### 5.4. Selection Within a Trial

Once we have specified the trial budget and depth rules from a particular root state, we have to decide how to actually select the actions and states that will appear in each individual trial (they may unroll in different directions). In other words, we have specified the overall shape of all trials in Figure 4, but not yet how this shape will actually be unfolded. We will first discuss *action selection*, which happens in Algorithm 1 line 16 and in many algorithms also at line 8, when we set the next root through forward sampling. Afterwards, we will discuss *next state selection*, which happens in line 26 of Algorithm 1. The considerations that we discuss for both topics are listed in the fourth row of Table 2.

**Action selection** The first approach to action selection is to pick actions in an *ordered* way, where we select actions *independently* of our interaction history with the MDP. Examples include uninformed search methods, such as iterative deepening. A downside of ordered action selection is that it may spend much time on states with lower value estimates, which typically makes it infeasible in larger problems. Most methods therefore try to prioritize actions in trials based on knowledge from previous trials. A first category of approaches prioritize actions based on their (current) value estimate, which we will call *value-based selection*. The cardinal example of value-based selection is *greedy* action selection, which repeatedly selects actions with the highest current value estimate. This is the dominant approach in the heuristic search literature (Hart et al., 1968; Nilsson, 1971; Barto et al., 1995; Hansen and Zilberstein, 2001), where an *admissible* heuristic may guarantee that greedy action selection will find the optimal solution.

**Figure 5:**
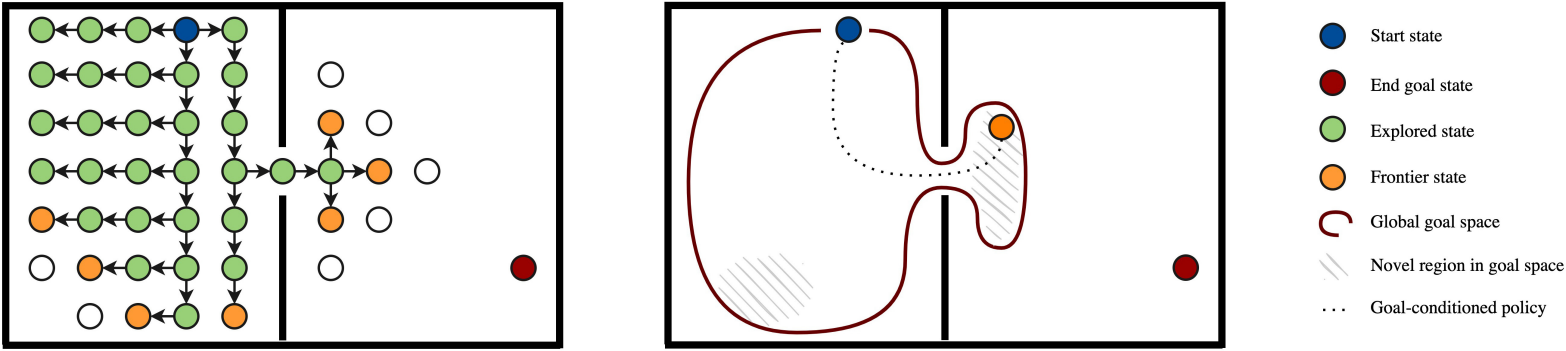
Frontier-based exploration in planning (left) and reinforcement learning (right, *intrinsic motivation*). Left: Frontier and explored set in a graph. Blue denotes the start state, red a final state, green denotes the explored set (states that have been visited and whose successors have been visited), orange denotes the frontier (states that have been visited but whose successors have not all been visited). Methods without a frontier and explored set (like random perturbation, which is used in most RL approaches) may sample many redundant trials that make loops in the left part of the problem, because they do not find the narrow passage. Right: In large problems, it may become infeasible to store the frontier and explored set in tabular form. Part of intrinsic motivation literature (Colas et al., 2020) tracks *global* (sub)goal spaces (red line) in global, approximate form. We may for example sample new goals from this space based on novelty, and subsequently attempt to reach that goal through a goal-conditioned policy, effectively mimicking frontier-based exploration in approximate, global form.

Note that heuristic search algorithms in practice usually maintain a *frontier* (Figure 5), and therefore do not actually need to greedily traverse the local solution toward the best leaf state. However, as Schulte and Keller (2014) also show, any ordering on the frontier can also be achieved by step-wise action selection from the root, and frontiers therefore conceptually fully fit into our framework (although the practical implementation may differ). The notion of frontiers is important, because algorithms that use a frontier often *switch* their action selection strategy once they reach the frontier. For example, a heuristic search algorithm may greedily select actions within the known part of the local solution, but at the frontier expand all possible actions, which is a form of ordered action selection. For some algorithms, we will therefore separately mention the action selection strategy *before the frontier* (BF) and *after the frontier* (AF).

Without an admissible heuristic greedy action selection is not guaranteed to find the optimal solution. Algorithms therefore usually introduce a form of *exploration*. A first option in this category is *random perturbation*, which is in the RL community usually referred to as ϵ-greedy exploration (Sutton and Barto, 2018). Similar ideas have been extensively studied in the planning community (Valenzano et al., 2014), for example in limited discrepancy search (Harvey and Ginsberg, 1995), *k*-best-first-search (KBFS) (Felner et al., 2003) and best-first width search (BFWS) (Lipovetzky and Geffner, 2017). We may also make the selection probabilities proportional to the current mean estimates of each action, which is for discrete and continuous action spaces for example achieved by Boltzmann exploration (Cesa-Bianchi et al., 2017) and entropy regularization (Peters et al., 2010).

A downside of random perturbation methods is their inability to naturally transition from exploration to exploitation. A solution is to track the uncertainty of value estimate of each action, i.e., *uncertainty-based perturbation*. Such approaches have been extensively studied in the multi-armed bandit literature (Slivkins, 2019), and successful exploration methods from RL and planning (Kaelbling, 1993; Kocsis and Szepesvári, 2006; Hao et al., 2019) are actually based on work from the bandit literature (Auer et al., 2002). Note that uncertainty estimation in sequential problems, like the MDP formulation, is harder than the multi-armed bandit setting, since we need to take the uncertainty in the value estimates of future states into account (Dearden et al., 1998; Moerland et al., 2017). As an alternative, we may also estimate uncertainty in a Bayesian way, and for example explore through Thompson sampling (Thompson, 1933; Osband et al., 2016). Note that *optimistic initialization* of the solution, already discussed Section 5.1, also uses optimism in the face of uncertainty to guide exploration, although it does not track the true uncertainty in the value estimates.

In contrast to value-based perturbation, we may also use *state-based perturbation*, where we inject exploration noise *based on our interaction history with the MDP* (i.e., independently of the extrinsic reward). As a classic example, a particular state might be interesting because it is novel, i.e., we have not visited it before in our current interaction history with the MDP. In the reinforcement learning literature, this approach is often referred to as *intrinsic motivation* (IM) (Chentanez et al., 2005; Oudeyer et al., 2007). We already encountered the same idea in the planning literature through the use of frontiers and explored set, which essentially prevent expansion of a state that we already visited before. In the RL (intrinsic motivation) literature, we usually make a separation between *knowledge-based* intrinsic motivation, which marks states or actions as interesting because they provide new knowledge about the MDP, and *competence-based* intrinsic motivation, where we prioritize target states based on our *ability* to reach them. Examples of the knowledge-based IM include intrinsic rewards for *novelty* (Brafman et al., 2003; Bellemare et al., 2016), recency (Sutton, 1990), curiosity (Pathak et al., 2017), surprise (Achiam and Sastry, 2017), and model uncertainty (Houthooft et al., 2016), while we may also provide intrinsic motivation for the *content* of a state, for example a saliency for objects (Kulkarni et al., 2016). Competence-based IM may for example prioritize (goal) states of intermediate difficulty (which we manage to reach sometimes) (Florensa et al., 2018), or on which we are currently making learning progress (Lopes et al., 2012; Baranes and Oudeyer, 2013; Matiisen et al., 2017).

As mentioned above, there is clear connection between the use of frontiers in planning literature and the use of intrinsic motivation in reinforcement learning literature, which we illustrate in Figure 5. On the one hand, the planning literature has many techniques to track and prioritize frontiers, but these tabular approaches do suffer in high-dimensional problems. In contrast, in RL methods that do not track frontiers (but for example use random perturbation) many trials may not hit a new state at all (Ecoffet et al., 2021). Intrinsic motivation literature has studied the use of *global, approximate frontiers* (i.e., global, approximate sets of interesting states to explore), which is typically referred to as intrinsically motivated goal exploration processes (IMGEP) (Colas et al., 2020). A successful example algorithm in this class is Go-Explore (Ecoffet et al., 2021), which achieved state-of-the-art performance on the sparse-reward benchmark task Montezuma's Revenge. However, IMGEP approaches have their challenges as well, especially because it is hard to track convergence of approximate solutions, and our goal space may for example be off, or we do encounter a novel region but after an update of our goal-conditioned policy we are not able to get back. Tabular solutions from the planning literature do not suffer from these issues, and we conjecture that there is much potential here in the combination of ideas from both research fields.

As mentioned in the beginning, action selection often also plays a role on Algorithm 1 line 8, when we select next root states through forward sampling from the previous root (as discussed in Section 5.2). In the planning literature, this is often referred to as the *recommendation function* (Keller and Helmert, 2013) (what action do we recommend at the root after all trials and back-ups). When we want to maximize performance, action recommendation is often greedy. However, during offline learning, we may inject additional exploration into action selection at the root, for example by *planning to explore* (the trials in a learned model direct the agent toward interesting new root state in the true environment) (Sekar et al., 2020). We will refer to this type of action selection as *next root* (NR) selection, and note that some algorithms therefore have three different action selection strategies: before the frontier (BF) within a trial, after the frontier (AF) within a trial, and to set the next root (NR) for new trials. An overview of the discussed action selection methods, with some characteristic examples, is provided in Table 4.

**Table 4:** Overview of action selection methodology within a trial.

**Action selection method**	**Characteristic examples**
**Ordered**	Value iteration Bellman, 1966 Iterative deepening Korf, 1985
**Value-based**	
- Greedy (with heuristic)	AO^⋆^ Nilsson, 1971
	RTDP Barto et al., 1995
- Random perturbation	ϵ-greedy Sutton and Barto, 2018
	Gaussian noise Van Hasselt and Wiering, 2007
- Mean perturbation	Boltzmann Cesa-Bianchi et al., 2017
	Entropy regularization Peters et al., 2010
- Uncertainty perturbation	Upper confidence bounds Kaelbling, 1993
	Posterior sampling Thompson, 1933
**State-based**	
- Knowledge-based IM	Novelty Brafman et al., 2003
	Suprise Achiam and Sastry, 2017
- Competence-based IM	Learning progress Péré et al., 2018
	Goal-reaching success Florensa et al., 2018

*At the highest level, we may either prioritized actions in an ordered way (independent of our interaction history with the MDP), in a value-based way (based on obtained rewards in our interaction history with the MDP), or in astate-based (based on our interaction history with the MDP, but independent of the value). The table shows possible subcategories, and some characteristic examples in the right column*.

**State selection** After our extensive discussion of action selection methods within a trial, we also need to discuss *next state selection*, which happens at line 26 of Algorithm 1. The two possible options here are ordered and sample selection. *Ordered* next state selection is for example used in value and policy iteration, where we simply expand every possible next state of an action. This approach is only feasible when we have settable, descriptive access to the MDP dynamics (see Section 3.2), since we can then decide ourselves which next state we want to make our next MDP query from. The second option is to *sample* the next state, which is by definition the choice when we only have generative access to the MDP dynamics. However, sampled next state selection may even be beneficial when we do have descriptive access (Sutton and Barto, 2018).

**Figure 6:**
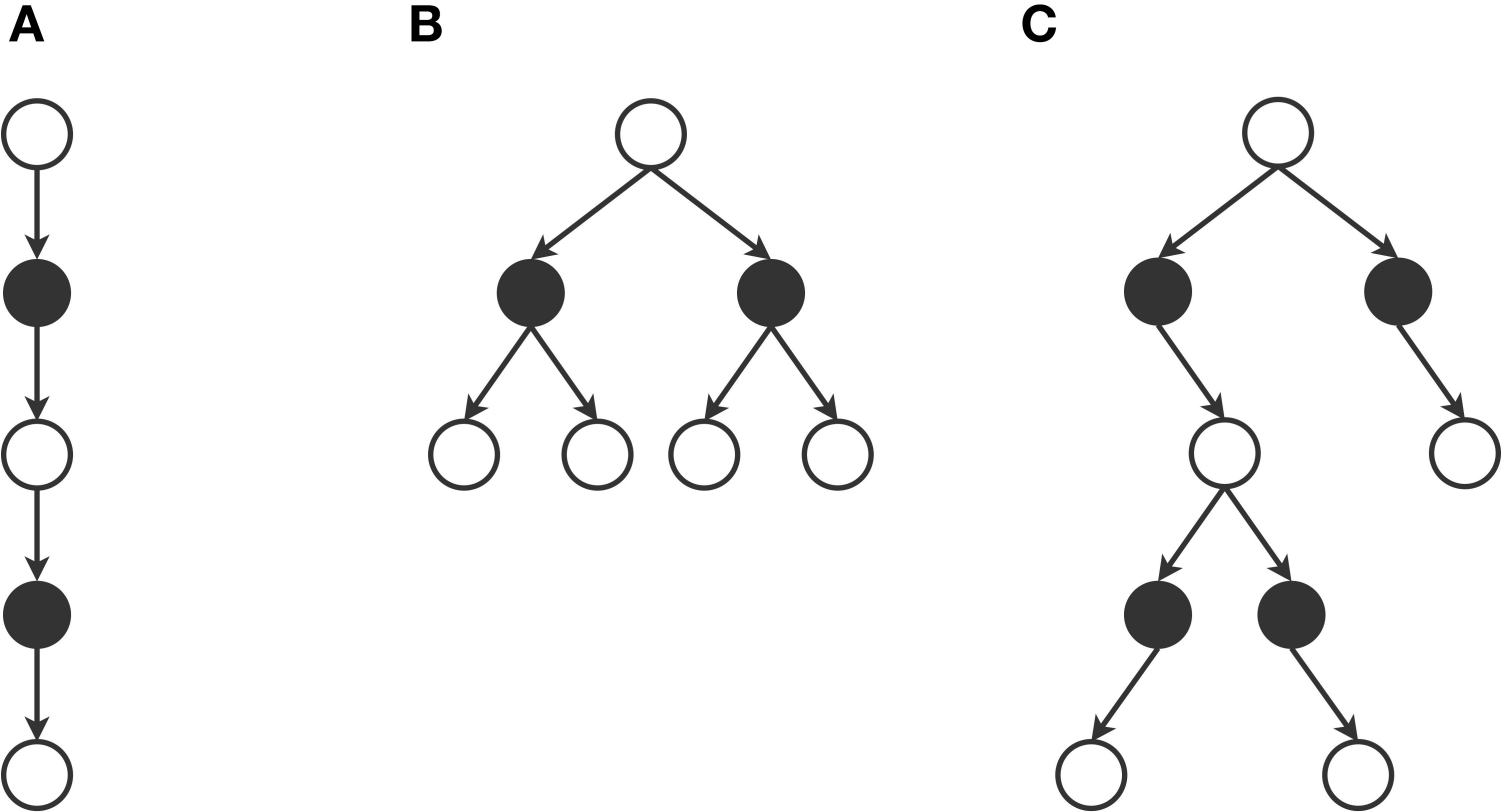
Example local solution patterns. **(A)** Local solution consisting of a single trial with depth 2. Total queries to the MDP = 2. Example: two-step temporal difference learning. **(B)** Local solution consisting of four trial with depth 1. Total queries to the MDP = 4. Example: value iteration. **(C)** Local solution consisting of three trials, one with depth 1 and two with depth 2. Total queries to the MDP = 4. Example: Monte Carlo Tree Search.

To summarize this section on action and next state selection within a trial, Figure 6 illustrates some characteristic trial patterns. On the left of the figure we visualize a local solution consisting of a single trial with *d*_max_ = 2, which is for example used in two-step temporal difference (TD) learning (Sutton, 1988). In the middle, we see a local solution consisting of four trials, each with a *d*_max_ of 1. Each action and next state is selected in an ordered way, which is for example used in value iteration (Bellman, 1966). Finally, the right side of the figure shows a local solution consisting of three trials, one with *d*_max_ = 1 and two with *d*_max_ = 2, which could for example appear in Monte Carlo Tree Search (Kocsis and Szepesvári, 2006). With the methodology described in this section, we can construct any other preferred local solution pattern. In the next section we will discuss what to do at the leaf states of these patterns, i.e., what to do when we reach the trial's *d*_max_.

### 5.5. Bootstrap

The main aim of trials is to provide a new/improved estimate of the value of each action at the root, i.e., the expected cumulative sum of rewards from this state-action (Equation 1). However, when we choose to end a trial before we can evaluate the entire sum, we may still obtain an estimate of the cumulative reward through *bootstrapping*. A bootstrap function is a function that provides a quick estimate of the value of a particular state or state-action. When we decide to end our trial at a state, we need to bootstrap a state value (Algorithm 1, line 14), and when we decide to end the trial at an action, we need to bootstrap a state-action value (Algorithm 1, line 23). A potential benefit of a state value function is that it has lower dimension and might be easier to learn/obtain, while a state-action value function has the benefit that it allows for off-policy back-ups (see Section 5.6) without additional queries to the MDP. Note that terminal states have a value of 0 by definition.

The bootstrap function itself may either be obtained from a *heuristic function*, or it can be learned. Heuristic functions have been studied extensively in the planning community. A heuristic is called *admissible* when it provides an *optimistic* estimate of the remaining value for every state, which allows for greedy action selection strategies during the search. Heuristics can be obtained from prior knowledge, but much research has focused on automatic ways to obtain heuristics, often by first solving a simplified version of the problem. When the problem is stochastic, a popular approach is *determinization*, where we first solve a deterministic version of the MDP to obtain a heuristic for the full planning task (Hoffmann and Nebel, 2001; Yoon et al., 2007), or *delete relaxations* (Bonet and Geffner, 2001), where we temporarily ignore the action effects that remove state attributes (which is only applicable in symbolic states spaces). A heuristic is called 'blind' when it is initialized to the same value everywhere. For an extensive discussion of ways to obtain heuristics we refer the reader to Pearl (1984) and Edelkamp and Schrodl (2011).

The alternative approach is to *learn* a global state or state-action value function. Note that this function can also serve as our solution representation (see Section 5.1). The learned value function can be trained on the root value estimates of previous trials (see Section 5.7), and thereby gradually improve its performance (Sutton, 1988; Korf, 1990). A major benefit of learned value functions is 1) their ability to improve performance with more data, and 2) their ability to *generalize* when learned in approximate form. For example, while Deep Blue (Campbell et al., 2002), the first computer programme to defeat a human Chess world champion, used a heuristic bootstrap function, this approach was later outperformed by AlphaZero (Silver et al., 2018), which uses a deep neural network to learn a bootstrap function that provides better generalization.

### 5.6. Back-Up

Bootstrapping ends the forward phase of a trial, after which we start the back-up phase (Figure 2, right). The goal of back-ups is to process the acquired information of the trial. We will primarily focus on the *value back-up*, where we construct new estimates $\hat{V}\left(s\right)$ and $\hat{Q}\left(s,a\right)$ for states and actions that appear in the trial. At the end of this section, we will also briefly comment on other types of information we may include in the back-up.

Value back-ups are based on the one-step Bellman equation, as shown in Equation 2. The first expectation of this equation, over the possible next states, shows the *dynamics back-up*: we need to aggregate value estimates for different possible next states into an state-action value estimate for the state-action that may lead to them. The second expectation, over the possible actions, shows the *policy back-up*: we want to aggregate state-action values into a value estimate at the particular state. We therefore need to discuss how to deal with width (expectations) over the policy and dynamics. In Algorithm 1, policy and dynamics back-ups happen at line 18 and 28, while we will now discuss the relevant considerations for these back-ups, as listed in the sixth row of Table 2.

For the policy back-up, we first need to specify which back-up policy we will actually employ. A first option is to use the current behavioral policy (which we used for action selection within the trial) as the back-up policy, which is in RL literature usually referred to as *on-policy* back-ups. An alternative is to use another policy than the behavioral policy, which is referred to as *off-policy*. The most common off-policy back-up is the *greedy* or *max* back-up, which puts all probability on the action with the highest current value estimate. The greedy back-up is common in tabular solutions, but can be unstable when combined with a global approximate solutions and bootstrapping (Van Hasselt et al., 2018). Note that off-policy back-ups do not need to be greedy, and we may also use back-up policies that are more greedy than the exploration policy, but less greedy than the max operator (Coulom, 2006; Keller, 2015).

We next need to decide whether we will make a *full*/*expected* policy back-up, or a *partial*/*sample* policy back-up. Expected back-ups evaluate the full expectation over the policy probabilities, and therefore need to expand all child actions of a state. In contrast, sample back-ups only back-up the value from a sampled action, and therefore do not need to trial all child actions (and are therefore called “partial”). Sample back-ups are less accurate but computationally cheaper, and will move toward the true value over multiple samples.

**Figure 7:**
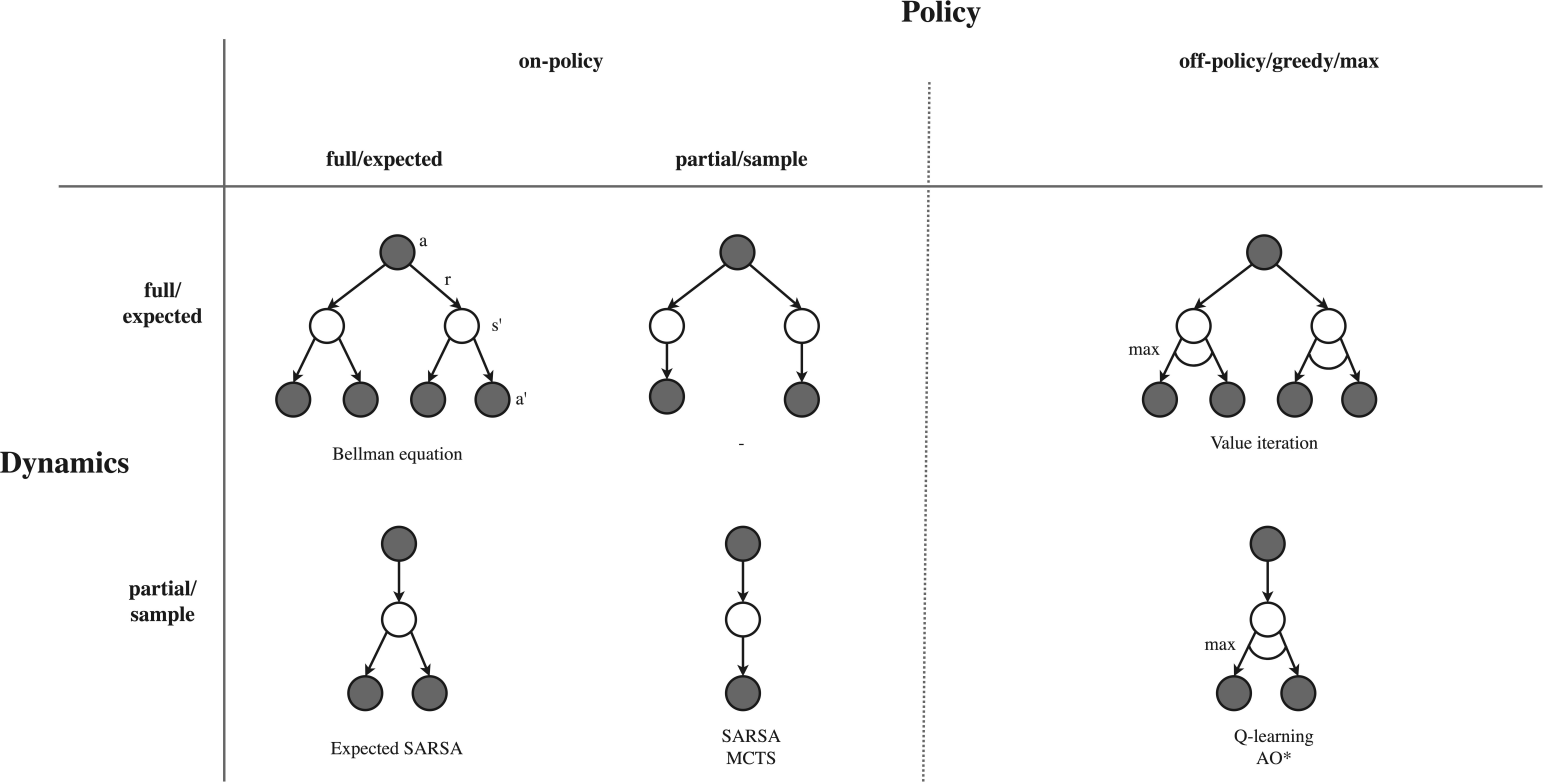
Types of 1-step back-ups. For the back-up over the policy (columns), we need to decide on i) the type of policy (on-policy or off-policy) and ii) whether we do a full or partial back-up. For the back-up over the dynamics (rows), we also need to decide whether we do a full or partial back-up. Note that for the greedy/max back-up policy the expected and sample back-ups are equivalent. Mentioned algorithms: Value Iteration (Bellman, 1966), Expected SARSA (Van Seijen et al., 2009), SARSA (Rummery and Niranjan, 1994), MCTS (Kocsis and Szepesvári, 2006), Q-learning (Watkins and Dayan, 1992), and AO^⋆^ (Nilsson, 1971).

The same consideration actually applies to the back-up over the dynamics, which can also be *full*/*expected* back-up, or *partial*/*sample*. Which type of dynamics back-up we can make also depends on the type of access we have to the MDP. When we only have generative access to the MDP, we are forced to make sample back-ups. In contrast, when we have descriptive access to the MDP, we can either make expected or sample back-ups. Although sample back-ups have higher variance, they are computationally cheaper and may be more efficient when many next states have a small probability (Sutton and Barto, 2018). We summarize the common back-up equations for policy and dynamics in Table 5, while Figure 7 visualizes common combinations of these as back-up diagrams.

**Table 5:** Equations for the policy and dynamics back-up, applicable to Algorithm 1 line 18 and 28, respectively.

	**Equation**	
**Policy**		
Sample back-up	$\hat{V}\left(s\right)\leftarrow \hat{Q}\left(s,a\right)$,	for *a*~π(·|*s*′)
Expected back-up	$\hat{V}\left(s\right)\leftarrow E_{a\sim \pi \left(\cdot |s\right)}\left[\hat{Q}\left(s,a\right)\right]$	
Greedy back-up	$\hat{V}\left(s\right)\leftarrow \underset{a}{\max}\left[\hat{Q}\left(s,a\right)\right]$	
**Dynamics**		
Sample back-up	$\hat{Q}\left(s,a\right)\leftarrow R\left(s,a,s^{\prime }\right)+\gamma \cdot \hat{V}\left(s^{\prime }\right)$,	for *s*′~*T*(·|*s, a*)
Expected back-up	$\hat{Q}\left(s,a\right)\leftarrow E_{s^{\prime }\sim T\left(s^{\prime }|s,a\right)}\left[R\left(s,a,s^{\prime }\right)+\gamma \cdot \hat{V}\left(s^{\prime }\right)\right]$	

Many algorithms back-up additional information to improve action selection in future trials. We may want to track the uncertainty in the value estimates, for example by backing-up visitation counts (Browne et al., 2012), by backing-up entire uncertainty distributions around value estimates (Dearden et al., 1998; Deisenroth and Rasmussen, 2011), or by backing-up the distribution of the return (Bellemare et al., 2017). Some methods back-up *labels* that mark a particular value estimate as “solved” when we are completely certain about its value estimate (Nilsson, 1971; Bonet and Geffner, 2003b). As mentioned before, graph searches also back-up information about frontiers and explored sets, which can be seen as another kind of label, one that removes duplicates and marks expanded states. The overarching theme in all these additional back-ups is that they track some kind of uncertainty about the value of a particular state, which can be utilized during action selection in future trials.

### 5.7. Update

The last step of the framework involves updating the local solutions (*V*^**l**^(*s*) and *Q*^**l**^(*s, a*)) based on the back-up estimates ($\hat{V}\left(s\right)$ and $\hat{Q}\left(s,a\right)$), and subsequently updating the global solution (*V*^**g**^(*s*) and/or *Q*^**g**^(*s, a*) and/or π^**g**^(*a*|*s*)) based on the local solution. In Algorithm 1, the updates of the local solution happen in lines 19 and 29, while the update of the global solution (when used) occurs in line 7. The main message of this section is that we can write both types of updates, whether it concerns updates of nodes in a planning tree or updates of a global policy network, as *gradient descent* updates on a particular *loss function*. We hope this provides further insight in the similarity between planning and learning, since planning updates on a tree/graph can usually be written as tabular learning updates with a particular learning rate.

We will first introduce our general notation. A loss function is denoted by L(θ), where θ denotes the parameters to be updated. In case of a tabular solution, the parameters are simply the individual entries in the table (like *Q*^**l**^(*s, a*))) (see Section 5.1 and Table 3 for a summary of notation), and we will therefore not explicitly add a subscript θ. When we have specified a solution and a loss function, the parameters can be updated based on gradient descent, with update rule:


(4) $$\begin{array}{l} \theta \leftarrow \theta -\eta \;\cdot \;\nabla_{\theta }ℒ(\theta ), \end{array}$$

where η∈ℝ^+^ is a learning rate. We will first show which loss function and update rules are common in updating of the local solution, and subsequently discuss how they reappear in updates of the global solution based on the local solution. An overview of common loss functions and update rules is provided in Table 6, which we will now discuss in more detail.

**Table 6:** Overview of common loss functions and update rules.

	**Loss**	**Update**
**Local update**		
*Value*		
Squared loss	$L\left(Q^{\text{l}}\left(s,a\right)|s,a\right)=\frac{1}{2}\hat{Q}\left(s,a\right)-Q^{\text{l}}\left(s,a\right){\;}^{2}$	$Q^{\text{l}}\left(s,a\right)\leftarrow Q^{\text{l}}\left(s,a\right)+\eta \cdot \left[\hat{Q}\left(s,a\right)-Q^{\text{l}}\left(s,a\right)\right]$
Replace update (η = 1)		$Q^{\text{l}}\left(s,a\right)\leftarrow \hat{Q}\left(s,a\right)$
Average update ($\eta =\frac{1}{n}$)		$Q^{\text{l}}\left(s,a\right)\leftarrow Q^{\text{l}}\left(s,a\right)+\frac{1}{n}\cdot \left[\hat{Q}\left(s,a\right)-Q^{\text{l}}\left(s,a\right)\right]$
Eligibility update		$Q^{\text{l}}\left(s,a\right)\leftarrow Q^{\text{l}}\left(s,a\right)+\left(1-\lambda \right)\cdot \lambda^{\left(d-1\right)}\cdot \left[{\hat{Q}}_{d}\left(s,a\right)-Q^{\text{l}}\left(s,a\right)\right]$
**Global update**		
*Value*		
Squared loss	$L\left(\theta |s,a\right)=\frac{1}{2}Q^{\text{l}}\left(s,a\right)-Q_{\theta }^{\text{g}}\left(s,a\right){\;}^{2}$	$\theta \leftarrow \theta +\eta \cdot \left[Q^{\text{l}}\left(s,a\right)-Q_{\theta }^{\text{g}}\left(s,a\right)\right]\cdot \nabla_{\theta }Q_{\theta }^{\text{g}}\left(s,a\right)$
Cross-entropy softmax loss	$L\left(\theta |s\right)=-\text{softmax}{\left(Q^{\text{l}}\left(s,\text{a}\right)\right)}^{T}\cdot \log\text{softmax}\left(Q_{\theta }^{\text{g}}\left(s,\text{a}\right)\right)$	$\theta \leftarrow \theta +\eta \cdot \nabla_{\theta }[\text{softmax}{\left(Q^{\text{l}}\left(s,\text{a}\right)\right)}^{T}\cdot \log\text{softmax}\left(\left(Q_{\theta }^{\text{g}}\left(s,\text{a}\right)\right)\right]$
*Policy*		
Policy gradient	$L\left(\theta |s,a\right)=-\ln\;\pi_{\theta }^{\text{g}}\left(a|s\right)\cdot Q^{\text{l}}\left(s,a\right)$	$\theta \leftarrow \theta +\eta \cdot \frac{Q^{\text{l}}\left(s,a\right)}{\pi_{\theta }^{\text{g}}\left(a|s\right)}\cdot \nabla_{\theta }\pi_{\theta }^{\text{g}}\left(a|s\right)$
Determ. policy gradient	$L\left(\theta |s,a\right)=-Q_{\psi }^{\text{g}}\left(s,\pi_{\theta }^{\text{g}}\left(a|s\right)\right)$ ($Q_{\psi }^{\text{g}}$ trained on *Q*^**l**^)	$\theta \leftarrow \theta +\eta \cdot \nabla_{a}Q_{\psi }^{\text{g}}\left(s,a\right)\cdot \nabla_{\theta }\pi_{\theta }^{\text{g}}\left(a|s\right)$
Value gradient	$L\left(\theta |s\right)=-V^{\text{l}}\left(s\right)$	$\theta \leftarrow \theta +\eta \cdot \nabla_{\theta }V^{\text{l}}\left(s\right)$ (Figure 8)
Cross-entropy loss	$L\left(\theta |s\right)=\underset{a\in A}{\sum }\ln\;\pi_{\theta }\left(a|s_{t}\right)\frac{n^{\text{l}}\left(s_{t},a\right)}{\underset{b\in A}{\sum }n^{\text{l}}\left(s_{t},b\right)}$	$\theta \leftarrow \theta -\eta \cdot \underset{a\in A}{\sum }\frac{n^{\text{l}}\left(s_{t},a\right)}{\underset{b\in A}{\sum }n^{\text{l}}\left(s_{t},b\right)}\cdot \frac{1}{\pi_{\theta }^{\text{g}}\left(a|s\right)}\cdot \nabla_{\theta }\pi_{\theta }^{\text{g}}\left(a|s\right)$

*Top: Local update, where we use back-up values $\hat{V}\left(s\right)$ and/or $\hat{Q}\left(s,a\right)$ to update the local solution V^**l**^(s) and/or Q^**l**^(s, a). The special cases of replace update and average update are explicitly shown. Bottom: Global update, where we use the local solution estimates V^**l**^(s) and/or Q^**l**^(s, a) to update global (approximate) solutions $V_{\theta }^{\text{g}}\left(s\right)$, $Q_{\theta }^{\text{g}}\left(s,a\right)$ and/or $\pi_{\theta }^{\text{g}}\left(a|s\right)$. Parameters of the global solution are denoted by θ (when the global value solution is tabular each θ in the table can be read as Q^**g**^(s, a)). Note that the table illustrates some characteristic examples, but other losses and update rules are possible. ${\hat{Q}}_{d}\left(s,a\right)$ denotes an estimate from a trial of depth d*.

**Local solution update** Here we will focus on the update of state-action values *Q*^**l**^(*s, a*) (Algorithm 1, line 29), but the same principles apply to state value updating (Algorithm 1, line 19). We therefore want to specify an update of *Q*^**l**^(*s, a*) based on a new back-up value $\hat{Q}\left(s,a\right)$. A classic choice of loss function for continuous values is the *squared loss*, given by:


(5) $$\begin{array}{l} ℒ(Q^{l}(s,a)|s,a)=\frac{1}{2}{\left[\hat{Q}(s,a)-Q^{l}(s,a)\right]}^{2}. \end{array}$$

Differentiating this loss with respect to *Q*^**l**^(*s, a*) and plugging it into Equation (4) (where *Q*^**l**^(*s, a*) are the parameters) gives the well-known *tabular learning rule*:


(6) $$\begin{array}{l} Q^{l}(s,a)\leftarrow Q^{l}(s,a)+\eta \cdot [\hat{Q}(s,a)-Q^{l}(s,a)]. \end{array}$$

Intuitively, we move our estimate *Q*^**l**^(*s, a*) a bit in the direction of our new back-up value $\hat{Q}\left(s,a\right)$. In the tabular case, η is therefore restricted to [0, 1]. Most planning algorithms use special cases of the above update rule. A first common choice is to set η = 1.0, which gives the *replace update*:


(7) $$\begin{array}{r} Q^{\text{l}}\left(s,a\right)\leftarrow \hat{Q}\left(s,a\right). \end{array}$$

This update completely overwrites the estimate in the local solution by the new back-up value. This is the typical approach in heuristic planning (Hart et al., 1968; Nilsson, 1971; Hansen and Zilberstein, 2001), where an admissible heuristic often ensures that our new estimate (from a deeper unfolding of the planning tree) provides a better informed estimate than the previous estimate. Although one would typically not think of such a replace update as a gradient-based approach, these updates are in fact all connected.

When we do not have a good heuristic available (and we therefore need to bootstrap from a learned value function or use deep roll-outs to estimate the cumulative reward), estimates of different depths may have different reliability (known as the *bias-variance trade-off* ) (Sutton and Barto, 2018). We may for example equally weight the contribution of estimates of different depths, which we will call an *averaging update* (which uses $\eta =\frac{1}{n}$, where *n* denotes the number of trials/back-up estimates for the node):


(8) $$\begin{array}{r} Q^{\text{l}}\left(s,a\right)\leftarrow Q^{\text{l}}\left(s,a\right)+\frac{1}{n}\cdot \left[\hat{Q}\left(s,a\right)-Q^{\text{l}}\left(s,a\right)\right] \end{array}$$

This is for example used in MCTS implementations that use bootstrapping instead of rollouts (Silver et al., 2018).

While the above update gives the value estimate from each trial equal weight, we may also make the contribution of a trial estimate dependent on the depth of the trial, as is for example done in *elegibility traces* (Schulman et al., 2016; Sutton and Barto, 2018). In this case, we essentially set η = (1−λ)·λ^(*d*−1)^, where λ∈[0, 1] is the exponential decay and *d* is the length of the trace on which we update. More sophisticated reweighting schemes of the targets of different trials are possible as well (Munos et al., 2016), for example based on the *uncertainty* of the estimate at each depth (Buckman et al., 2018). In short, the local solution may combine value estimates from different trials (with different depths) in numerous ways, as summarized in the top part of Table 6.

**Global solution update** When our algorithm uses a global solution, we next need to update this global solution (*V*^**g**^ and/or *Q*^**g**^ and/or π^**g**^) based on the estimates from our local solution (*V*^**l**^ and/or *Q*^**l**^) (Algorithm 1, line 7). For a value-based solution that is *tabular*, we typically use the same squared loss (Equation 5), which leads to the global tabular update rule *Q*^**g**^(*s, a*)←*Q*^**g**^(*s, a*)+η·[*Q*^**l**^(*s, a*)−*Q*^**g**^(*s, a*)], which exactly resembles the local version (Equation 6), apart from the fact that we now update *Q*^**g**^(*s, a*), while *Q*^**l**^(*s, a*) has the role of target. This approach is the basis of all tabular RL methods (Sutton and Barto, 2018). [For (model-free) RL approaches that directly update the global solution after a single trial, we may also imagine the local solution does not exist, and we directly update the global solution from the back-up estimates].

We will therefore primarily focus on the function approximation setting, where we update a global approximate representation parametrized by θ. Table 6 shows some example loss functions and update rules that appear in this case. The most important point to note is that there are many ways in which we may combine a local estimate, such as *Q*^**l**^(*s, a*), and the global solution, such as *Q*^**g**^(*s, a*) or π^**g**^(*a*|*s*), in a loss function. For value-based updating, we may use the squared loss, but other options are possible as well, like a cross-entropy loss over the softmax of the Q-values returned from planning (the local solution) and the softmax of the Q-values from a global neural network approximation (Hamrick et al., 2020a). For policy-based updating, well-known examples include the *policy gradient* (Williams, 1992; Sutton et al., 2000; Sutton and Barto, 2018) and *deterministic policy gradient* (Silver et al., 2014; Lillicrap et al., 2015) loss functions. Again, other options have been successful as well, such as a cross-entropy loss between the normalized visitations counts at the root of an MCTS (part of the local solution) and a global policy network, as for example used by AlphaZero (Silver et al., 2017). In short, various objectives are possible (and more may be discovered), as long as minimization of the objective moves our global solution in the right direction (based on the obtained information from the trial).

**Figure 8:**
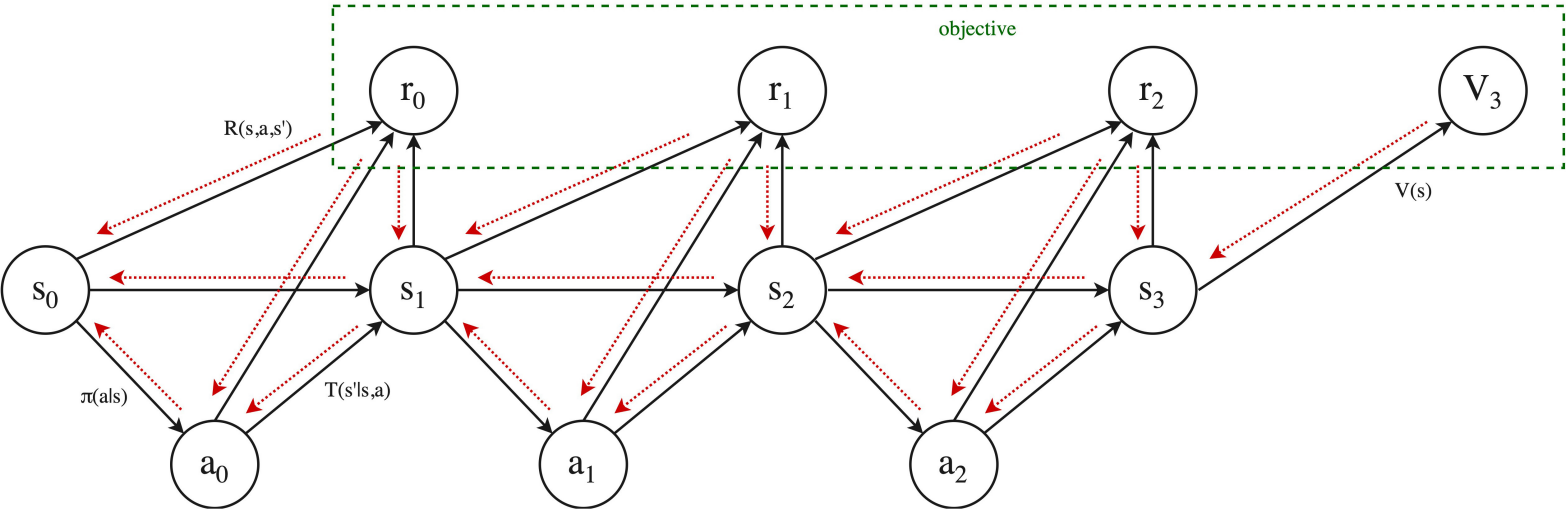
Illustration of gradient-based planning. When we have access to a differentiable transition function $T\left(s^{\prime }|s,a\right)$ and differentiable reward function $R\left(s,a,s^{\prime }\right)$, and we also specify a differentiable policy π_θ_(*a*|*s*), then a single trial generates a fully differentiable computational graph. The figure shows an example graph for a trial of depth 3. The black arrows show the forward passes through the policy, dynamics function and rewards function. In the example, we also bootstrap from a differentiable (learned) value function, but this can also be omitted. We may then update the policy parameters by directly differentiating the cumulative reward (objective, green box) with respect to the policy parameters, effectively summing the gradients over all backwards path indicated by the red dotted lines.

An important other class of approaches is *gradient-based planning*, also known as *value gradients* (Fairbank and Alonso, 2012; Heess et al., 2015). These approaches require a (known or learned) differentiable transition and reward model (and a differentiable value function when we also include bootstrapping). When we also specify a differentiable policy, then each trial generates a fully differentiable graph, in which we can directly differentiate the cumulative reward with respect to the policy parameters. This idea is illustrated in Figure 8, where we aggregate over all gradient paths in the graph (red dotted lines). Gradient-based planning is popular in the robotics and control community (Todorov and Li, 2005; Anderson and Moore, 2007; Deisenroth and Rasmussen, 2011), where dynamics functions are relatively smooth and differentiable, although the idea can also be applied with discrete states (Wu et al., 2017).

Table 6 summarizes some of the common loss functions we discussed. The examples in the table all have analytical gradients, but otherwise we may always use finite differences to numerically estimate the gradient of an objective. The learning rate in these update equations is typically tuned to a specific value (or decay scheme), although there are more sophisticated approaches that bound the step size, such as proximal policy optimization (PPO) (Schulman et al., 2017). Moreover, we did not discuss gradient-free updating of a global solution, because these algorithms typically do not exploit MDP-specific knowledge (i.e, they do not construct and back-up value estimates at states throughout the MDP, but only sample the objective function based on traces from the root). However, we do note that gradient-free black-box optimization can also be successful in MDP optimization, as for example show for evolutionary strategies Moriarty et al. (1999); Whiteson and Stone (2006); Salimans et al. (2017), simulated annealing (Atiya et al., 2003) and the cross-entropy method Mannor et al. (2003).

This concludes our discussion of the dimensions in the framework. An overview of all considerations and their possible choices is shown in Table 2, while Algorithm 1 shows how all these considerations piece together in a general algorithmic framework. To illustrate the validity of the framework, the next section will analyze a variety of planning and RL methods along the framework dimensions.

## 6. Comparison of Algorithms

Having discussed all the dimensions of the framework, we will now zoom out and reflect on its use and potential implications. The main point of our framework is that MDP planning and reinforcement learning algorithms occupy the same solution space. To illustrate this idea, Table 7 shows for a range of well-known planning (blue), model-free RL (red) and model-based RL (green) algorithms the choices they make on the dimensions of the framework. The list is of course not complete (we could have included any other preferred algorithm), but the table illustrates that the framework is applicable to a wide range of algorithms.

**Table 7:** Comparison of algorithms (columns) along the framework dimensions (rows).

**Dimension**	**Consideration**	Value iteration Bellman, 1966	LAO^⋆^ Hansen and Zilberstein, 2001	Labeled RTDP Bonet and Geffner, 2003b	Monte Carlo search Tesauro and Galperin, 1997	MCTS Kocsis and Szepesvári, 2006	Q-learning Watkins and Dayan, 1992	TD(λ) Sutton and Barto, 2018
MDP access		Settable descriptive	Settable descriptive	Settable descriptive	Settable generative	Settable generative	Resettable generative	Resettable generative
Solution	- Coverage	Global	Local	Local	Local	Local	Global	Global
	- Type	*V*(*s*)	*V*(*s*)	*V*(*s*)	*Q*(*s, a*)	*Q*(*s, a*)	*Q*(*s, a*)	*V*(*s*)
	- Method	Tabular	Tabular	Tabular	Tabular	Tabular	Tabular	Tabular
	- Initialization	Uniform	Heuristic	Heuristic	Uniform	Optimistic	Uniform	Uniform
Root	- Selection	Ordered	Forward sampling	Forward sampling	Forward sampling	Forward sampling	Forward sampling	Forward sampling
Budget	- # trials per root	up to |A|·|S|	till convergence	up to |A|·|S|	*n*	*n*	1	*d*_max_
	- Depth	1	1..*n*	1	∞	∞	1	1..*d*_max_
Selection	- Next action	Ordered	BF: Greedy, AF: Ordered, NR: Greedy	BF: Greedy, AF: Ordered, NR: Greedy	BF: Ordered AF: Baseline	BF: Uncertainty AF: Baseline NR: Greedy	Random pert.	Random pert.
	- Next state	Ordered	Ordered	Sample	Sample	Sample	Sample	Sample
Bootstrap	- Location	State	State	State	-	-	State-action	State
	- Type	Learned	Heuristic	Heuristic	-	-	Learned	Learned
Back-up	- Back-up policy	Greedy/max	Greedy/max	Greedy/max	On-policy	On-policy	Greedy/max	On-policy
	- Policy exp.	-	-	-	Sample	Sample	-	Sample
	- Dynamics exp.	Expected	Expected	Expected	Sample	Sample	Sample	Sample
	- Add. back-ups	-	Convergence label	Convergence label	-	Counts	-	-
Update	- Loss	(Squared)	(Squared)	(Squared)	(Squared)	(Squared)	(Squared)	(Squared)
	- Update type	Replace (η = 1.0)	Replace (η = 1.0)	Replace (η = 1.0)	Average (η = 1/*n*)	Average (η = 1/*n*)	Fixed step	Eligibility
**Dimension**	**Consideration**	REINFORCE Williams, 1992	DQN Mnih et al., 2015	Prioritized sweeping Moore and Atkeson, 1993	Dyna Sutton, 1990	PILCO Deisenroth and Rasmussen, 2011	AlphaGo Silver et al., 2017	Go-Explore (policy-based) Ecoffet et al., 2021
MDP access		Resettable generative	Resettable generative	Resettable generative	Resettable generative	Resettable generative	Settable generative	Resettable generative
Solution	- Coverage	Global	Global	Global	Global	Global	Global	Global
	- Type	π(*a*|*s*)	*Q*(*s, a*)	*Q*(*s, a*)	*Q*(*s, a*)	π(*a*|*s*)	π(*a*|*s*), *V*(*s*)	π(*a*|*s, g*), *V*(*s*)
	- Method	Tabular	Approximate (NN)	Tabular	Tabular	Approximate (GP)	Approximate (NN)	Approximate (NN)
	- Initialization	Uniform	Random	Uniform	Uniform	Random	Random	Random
Root	- Selection	Forward	Forward	Forward + backward	Forward + visited states	Forward	Forward	Forward
Budget	- # trials per root	1	1	1	1	1	1600	*d*_max_
	- Depth	∞	1	1	1	∞	MCTS: 1..*n* NR: ∞	1..*d*_max_
Selection	- Next action	Rand. pert. (stoch. policy)	Rand. pert. (ϵ-greedy)	State-based (novelty)	State-based (novelty) + Mean pert. (Boltzmann)	Rand. pert. (stoch. policy)	BF/AF: Uncertainty NR: Rand. pert.	BF: Novelty + Mean pert. (entropy), AF: Rand. pert.
	- Next state	Sample	Sample	Sample	Sample	Sample	Sample	Sample
Bootstrap	- Location	-	State-action	State-action	State-action	-	State	State
	- Type	-	Learned	Learned	Learned	-	Learned	Learned
Back-up	- Back-up policy	On-policy	Max/greedy	Max/greedy	On-policy	On-policy	On-policy	On-policy
	- Policy exp.	Sample	-	Max	Sample	Sample	Sample	Sample
	- Dynamics exp.	Sample	Sample	Expected	Sample	Sample	Sample	Sample
	- Add. back-ups	-	-	Priorities, counts	Counts	Uncertainty	Counts	Counts
Update	- Loss	Policy gradient	Squared	(Squared)	(Squared)	Value gradient	Cross-entropy (policy) + squared (value)	Policy gradient (PPO) + squared (value)
	- Learning rate	Fixed step	Fixed step	Fixed step	Fixed step	Fixed step	Local: Average Global: fixed step	Local: eligibility Global: adaptive

*Blue, red and green color denote planning, model-free RL and model-based RL algorithms, respectively (although Value Iteration is technically model-based RL under our definitions in Section 3, we still list it as first entry since it is a core algorithm). All methods that use a global solution also use a local solution (which we did not explicitly write in the table). Regarding action selection, when applicable we discriminate before frontier (BF) action selection, after frontier (AF) action selection, and next root (NR) action selection. When the squared loss is written between brackets, it means that the algorithm uses a direct tabular update rule and the squared loss is therefore never explicitly part of the algorithm. NN, neural network; GP, Gaussian Process, PPO = Proximal Policy Optimization (Schulman et al., 2017)*.

A first observation from the table is that it reads like a patchwork. On most dimensions the same decisions appear in both the planning and reinforcement learning literature, showing that both fields actually have quite some overlap in developed methodology. For example, the depth and back-up schemes of MCTS (Kocsis and Szepesvári, 2006) and REINFORCE (Williams, 1992) are exactly the same, but they differ in their solution coverage (MCTS only uses a local solution, REINFORCE updates a global solution after every trial) and exploration method. Such comparisons provide insight into the overlap and differences between various approaches.

The second observation of the table is therefore that *all algorithms have to make a decision on each dimension*. Therefore, even though we often do not consciously consider each of the dimensions when we come up with a new algorithm, we are still implicitly making a decision on each of them. The framework could thereby potentially help to structure the design of new algorithms, by consciously walking along the dimensions of the framework. It also shows what we should actually report about an algorithm to fully characterize it.

There is one deeper connection between planning and tabular reinforcement learning we have not discussed yet. In our framework, we treated the back-up estimates generated from a single model-free RL trial as a local solution. This increases consistency (i.e., allows for the pseudocode of Algorithm 1), but we could also view model-free RL as a direct update of the global solution based on the back-up estimate (i.e., skip the local solution). With this view we see another relation between common planning and tabular learning algorithms, such as MCTS (planning) and Monte Carlo reinforcement learning (MCRL). Both these algorithms sample trials and compute back-up estimates in the same way, but MCTS writes these to a local tabular solution (with learning rate $\eta =\frac{1}{n}$), while MCRL writes these to a global tabular solution (with fixed learning rate η). These algorithms from different research fields are therefore strongly connected, not only in their back-up, but also in their update schemes.

We will briefly emphasize elements of the framework, or possible combinations of choices, that could deserve extra attention. First of all, the main success of reinforcement learning originates from its use of global, approximate representations (Silver et al., 2017; Ecoffet et al., 2021), for example in the form of deep neural networks. These approximate representations allow for generalization between similar states, and planning researchers may therefore want to emphasize global solution representations in their algorithms. The other way around, a main part of the success of planning literature comes from the stability and guarantees of building local, tabular solutions. Combinations of both approaches show state-of-the-art results (Levine and Abbeel, 2014; Silver et al., 2017; Hamrick et al., 2020a), and each illustrate that we can be very creative in the way learned global solutions can guide new planning iterations, and the way planning output may influence the global solution and/or action selection. Important research questions are therefore how action selection within a trial can be influenced by the global solution (Algorithm 1, line 16), how a local solution should influence the global solution (i.e., variants of loss functions, Algorithm 1, line 7), and how we may adaptively assign planning budgets per root state (Algorithm 1, line 5). A recent systematic study of design considerations in planning in the context of model-based deep reinforcement learning is provided by Hamrick et al. (2020b).

Another important direction for cross-pollination is the study of *global, approximate frontiers*. On the one hand, planning research has extensively studied the benefit of local, tabular frontiers, a crucial idea which has been ignored in most RL literature. On the other hand, tabular frontiers do not scale to high-dimensional problems, and in these cases we need to track some kind of global approximate frontier, as studied in intrinsically motivated goal exploration processes (Colas et al., 2020). Initial results in this direction are for example provided by Péré et al. (2018) and Ecoffet et al. (2021), but there appears to be much remaining research in this field. Getting back to the previous point, we also believe semi-parametric memory and episodic memory (Blundell et al., 2016; Pritzel et al., 2017) may play a big role for global approximate solutions, for example to ensure we can directly get back to a recently discovered interesting state.

A third interesting direction is a stronger emphasis on the idea of backward search (planning terminology) or prioritized sweeping (RL terminology). In both communities, backward search has received considerable less attention than forward search, while backward approaches are crucial to spread acquired information efficiently over a (global) state space (by setting root states in a smarter way, see Section 5.2). The major bottleneck seems the necessity of a *reverse* model (which state-actions may lead to a particular state), which is often available in smaller, tabular problems, but not in large complex problems where we only have a simulator or real world interaction available. However, we may learn an approximate reverse model from data, which could bring these powerful ideas back into the picture. Initial (promising) results in this direction are provided by Corneil et al. (2018), Edwards et al. (2018), and Agostinelli et al. (2019).

In summary, the framework for reinforcement learning and planning (FRAP), as presented in this paper, shows that both planning and reinforcement learning algorithms share the same algorithmic space. This provides a common language for researchers from both fields, and may help inspire future research (for example by cross-pollination). Finally, we hope the paper also serves an educational purpose, for researchers from one field that enter into the other, but particularly for students, as a systematic way to think about the decisions that need to be made in a planning or reinforcement learning algorithm, and as a way to integrate algorithms that are often presented in disjoint courses.

## Data Availability Statement

The original contributions presented in the study are included in the article/supplementary material, further inquiries can be directed to the corresponding author.

## Author Contributions

TM led the project and wrote the first manuscript. JB was involved in the conceptual design of the paper and provided feedback on the manuscript. AP and CJ both supervised the project, were involved in conceptual discussions, and provided comments on the manuscript to reach the final version. All authors contributed to the article and approved the submitted version.

## Conflict of Interest

The authors declare that the research was conducted in the absence of any commercial or financial relationships that could be construed as a potential conflict of interest.

## Publisher's Note

All claims expressed in this article are solely those of the authors and do not necessarily represent those of their affiliated organizations, or those of the publisher, the editors and the reviewers. Any product that may be evaluated in this article, or claim that may be made by its manufacturer, is not guaranteed or endorsed by the publisher.
